# Mechanistic investigations on six bacterial terpene cyclases

**DOI:** 10.3762/bjoc.12.173

**Published:** 2016-08-15

**Authors:** Patrick Rabe, Thomas Schmitz, Jeroen S Dickschat

**Affiliations:** 1 Kekulé-Institute of Organic Chemistry and Biochemistry, University of Bonn, Gerhard-Domagk-Straße 1, 53121 Bonn, Germany

**Keywords:** absolute configuration, biosynthesis, enzyme mechanisms, structure elucidation, terpenes

## Abstract

The products obtained by incubation of farnesyl diphosphate (FPP) with six purified bacterial terpene cyclases were characterised by one- and two-dimensional NMR spectroscopic methods, allowing for a full structure elucidation. The absolute configurations of four terpenes were determined based on their optical rotary powers. Incubation experiments with ^13^C-labelled isotopomers of FPP in buffers containing water or deuterium oxide allowed for detailed insights into the cyclisation mechanisms of the bacterial terpene cyclases.

## Introduction

Terpenes are structurally fascinating natural products with interesting molecular properties. Particularly obvious is their often characteristic aroma that contributes to the typical flavour of many essential oils from plants. This is exemplified by the monoterpene α-pinene that occurs in form of both enantiomers in pine trees, while (*R*)-(+)-limonene is found in citrus fruits. Cineol is one of the main constitutents of eucalyptus oil and (+)-carvone is present in caraway. Some famous sesquiterpenes are α-humulene from hops, α-patchoulene from patchouli oil, and β-cedrene from juniper [1]. Only a minority of terpenes such as cineol and α-humulene are achiral, and an interesting aspect is the observation that the two enantiomers of chiral terpenes can have very different smells, e.g., the (+)-enantiomer of carvone smells like caraway, while (−)-carvone occurs in spearmint and has a clear spearmint odor [2].

Odoriferous terpenes from bacteria were identified much later, with the first described compounds being geosmin [3] and 2-methylisoborneol [4], two irregular terpenoids that represent a degraded sesquiterpene [5] and a methylated monoterpene [6], respectively. These compounds have a musty or earthy aroma and are responsible for the smell of freshly ploughed earth. Only recent research revealed that terpenes are in fact widespread in bacteria, in particular in actinomycetes and a few other taxa that are actively engaged in secondary metabolism [7–9].

Since a couple of years the rapidly evolved genome sequencing techniques allow for a mining of terpene cyclases from bacterial genomes. Altogether, a number of ca. 1000 terpene cyclase genes are found in the genomes of sequenced bacteria [10], and about 50 bacterial terpene cyclases have so far been characterised for their products [11–31]. Due to the rapidly increasing number of sequenced bacterial genomes also the number of uncharacterised bacterial terpene cyclases grows fast. Therefore, we have recently developed a screening approach for the characterisation of bacterial terpene cyclases that is based on gene cloning by homologous recombination in yeast, followed by the heterologous expression in *Escherichia coli*, direct headspace sampling using a closed-loop stripping apparatus (CLSA) and compound identification by GC–MS [32–33]. Here we report on the purification of six of these bacterial sesquiterpene cyclases, purification and full structure elucidation of their products by NMR and determination of optical rotary powers. Furthermore, the enzyme mechanisms of the investigated terpene cyclases were studied by isotopic labelling experiments [34] similar to recently reported investigations on other bacterial [28,35] and fungal [36–37] terpene cyclases.

## Results and Discussion

**Figure 1:**
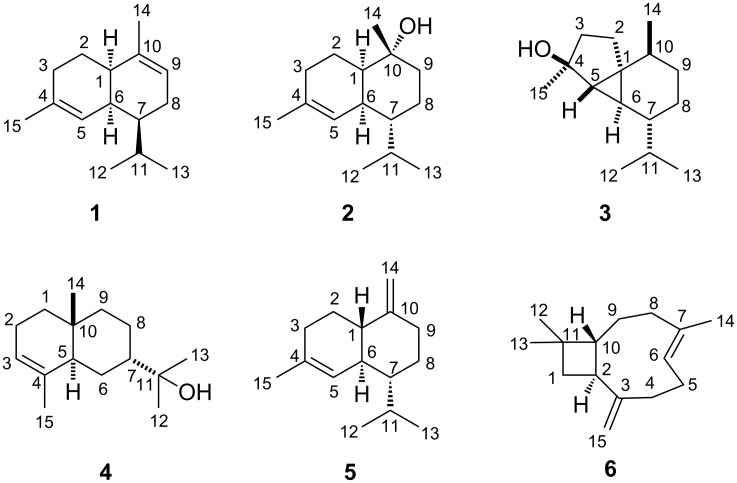
Structures of sesquiterpenes obtained by incubation of FPP with bacterial sesquiterpene cyclases.

**Figure 2:**
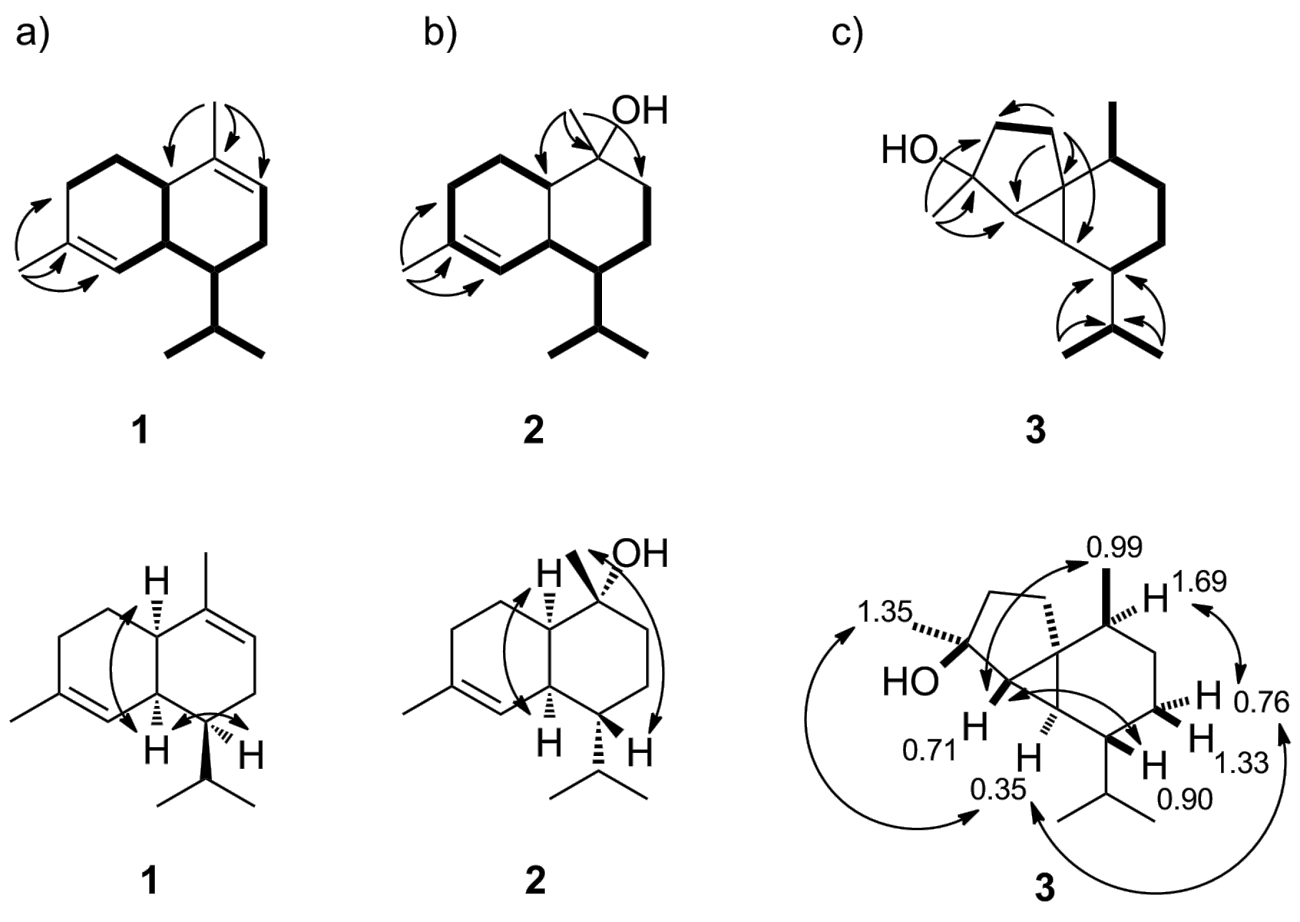
Contiguous spin systems observed by ^1^H,^1^H-COSY (bold), key HMBC and NOE correlations for a) α-amorphene (**1**), b) T-muurolol (**2**), and c) 4-*epi*-cubebol (**3**). ^1^H NMR shifts are given in ppm.

Incubation of a recombinant terpene cyclase from *Streptomyces viridochromogenes* DSM 40736 (NCBI accession number WP_039931950) with farnesyl diphosphate (FPP) yielded a single product that was identified as α-amorphene (**1**, Figure 1) by GC–MS analysis (Figure S1, Supporting Information File 1) [32], while the enzyme incubations with geranyl diphosphate (GPP) and geranylgeranyl diphosphate (GGPP) gave no products. Although **1** was isolated from vetiver oil (*Vetiveria zizanioides*, Gramineae) nearly five decades ago [38], the full set of assigned ^1^H and ^13^C NMR data has never been reported. The ^13^C NMR spectrum together with the ^13^C-DEPT135 spectrum exhibited signals for four methyl groups (CH_3_), three methylene groups (CH_2_), six methine (four sp^3^-CH and two sp^2^-CH) and two quarternary carbons (sp^2^-C), in agreement with a bicyclic structure (Table 1). The signals of the hydrogens attached to each carbon were assigned by HSQC spectroscopy, while the ^1^H,^1^H-COSY revealed a contiguous spin system C3-2-1-6(-5)-7(-11(-13)-12)-8-9 and two separate methyl groups attached to the quarternary olefinic carbons (Figure 2a). The HMBC spectrum showed key correlations between H-14 and C-1, C-9 and C-10, and between H-15 and C-3, C-4 and C-5, establishing the complete assignment of the carbon backbone. Key signals in the NOESY spectrum confirmed the relative configuration of **1** including its *cis*-decalin system and the anti-orientation between the isopropyl group and H-6. The optical rotary power of [α]_D_^22^ = −44.9 (*c* 0.15, CH_2_Cl_2_) proved that (1*R*,6*S*,7*S*)-(−)-**1** from *S. viridochromogenes* is the opposite enantiomer as in vetiver oil ([α]_D_ = +120) [38], while it is identical to the compound obtained by acid-catalysed rearrangement of (+)-α-ylangene [39]. The (−)-enantiomer of **1** has not been isolated as a natural product before.

**Table 1:** NMR data of α-amorphene (**1**), and T-muurolol (**2**) in (^2^H_6_)benzene.

C^a^	**1** ^1^H (δ, m, *J*)^b^	^13^C (δ)^b^	**2** ^1^H (δ, m, *J*)^b^	^13^C (δ)^b^

1	2.26 (br s, 1H)	39.0 (CH)	1.47 (m, 1H)	46.4 (CH)
2	1.59 (m, 1H) 1.93 (m, 1H)	25.7 (CH_2_)	1.43 (m, 2H)	21.3 (CH_2_)
3	1.61 (m, 1H) 1.90 (m, 1H)	26.8 (CH_2_)	1.87 (m, 2H)	31.6 (CH_2_)
4	–	136.0 (C_q_)	–	133.3 (C_q_)
5	5.29 (s, 1H)	120.0 (CH)	5.68 (d, *J* = 5.2, 1H)	125.8 (CH)
6	2.69 (br s, 1H)	36.5 (CH)	2.46 (m, 1H)	34.8 (CH)
7	1.15 (m, 1H)	45.4 (CH)	1.29 (m, 1H)	44.5 (CH)
8	1.67 (m, 1H) 1.99 (m, 1H)	27.6 (CH_2_)	1.28 (m, 1H) 1.50 (m, 1H)	19.8 (CH_2_)
9	5.48 (d, *J* = 3.7, 1H)	124.6 (CH)	1.28 (m, 1H) 1.43 (m, 1H)	35.0 (CH_2_)
10	–	133.4 (C_q_)	–	71.6 (C_q_)
11	1.56 (m, 1H)	29.1 (CH)	2.08 (dsept, *J* = 2.7, 6.9, 1H)	27.1 (CH)
12	0.94 (d, *J* = 6.7, 3H)	21.0 (CH_3_)	0.91 (d, *J* = 6.8, 3H)	21.9 (CH_3_)
13	0.89 (d, *J* = 6.7, 3H)	21.7 (CH_3_)	0.90 (d, *J* = 6.7, 3H)	15.7 (CH_3_)
14	1.64 (s, 3H)	20.9 (CH_3_)	1.05 (s, 3H)	29.6 (CH_3_)
15	1.64 (s, 3H)	24.3 (CH_3_)	1.64 (s, 3H)	23.9 (CH_3_)
OH	–	–	0.77 (br s, 1H, OH)	–

^a^Carbon numbering as in Figure 1. ^b^Chemical shifts δ in ppm; multiplicities: m = multiplet, s = singlet, d = doublet, sept = septet, br = broad; coupling constants *J* in Hz.

Type I terpene cyclases exhibit a few highly conserved motifs that are directly involved in binding of the Mg^2+^ cofactor to which in turn the substrate’s diphosphate portion is bound [10]. This includes the aspartate-rich (DDXXD) motif near position 90 and the NSE triad (ND(L,I,V)XSXXXE) around position 230 [40–41]. Furthermore, usually 46 positions upstream of the NSE triad a highly conserved arginine (pyrophosphate sensor) and a RY dimer near the C-terminus are found that form hydrogen bonds to the diphosphate moiety of the substrate [26]. Mutation within these conserved motifs is usually critical for enyzme functionality [26,41]. The (−)-α-amorphene synthase from *S. viridochromogenes* DSM 40736 displays the aspartate-rich motif (^105^DDRAE), the NSE triad (^242^PDLFSAVKE) starting with a proline instead of the usual asparagine, obviously without effect on the enzyme function, the pyrophosphate sensor R-196 and the ^327^RY dimer. Terpene cyclases with close homology are encoded in more than 40 genome sequenced streptomycetes that all show an altered NSE triad starting with either proline or alanine. The closest relative of the enzyme from *S. viridochromogenes* is found in *Streptomyces* sp. NRRL S-481 (86% identity).

Incubation of FPP with a recombinant terpene synthase from *Roseiflexus castenholzii* DSM 13941 (accession number WP_012119179) resulted in the formation of the sesquiterpene alcohol **2**, previously identified as T-muurolol by GC–MS [32], besides minor amounts of α-muurolene and δ-cadinene (Figure S1, Supporting Information File 1). The ^13^C NMR and ^13^C-DEPT135 spectra of purified **2** exhibited signals for four methyl groups, four methylene groups, five methine (four sp^3^-CH and one sp^2^-CH) as well as two quarternary carbons (the signal for C-10 with a chemical shift of δ = 71.6 ppm pointed to an attached hydroxy function, Table 1). HSQC spectroscopy provided information about the attached hydrogens and ^1^H,^1^H-COSY revealed one spin system C1-2-3-5-6-7(-11(-13)-12)-8-9, while the HMBC spectrum placed the two methyl groups C-14 and C-15 at the quarternary carbons C-10 and C-4, respectively (cross peaks between H-14 and C-1, C-9 and C-10 and between H-15 and C-3, C-4 and C-5, Figure 2b). Key NOE correlations between H-1 and H-6 confirmed the *cis*-decalin system, while NOE correlations of H-14 and H-7 finally established the structure of T-muurolol (**2**). The absolute configuration was determined as (1*R*,6*S*,7*R*,10*R*)-(+)-T-muurolol (**2**) from its optical rotary power ([α]_D_^23^ = +99.4 (*c* 1.10, CH_2_Cl_2_)). This is the same compound as was reported from a terpene cyclase from *Streptomyces clavuligerus* (accession number WP_003956090) [19], but the two enzymes show only a sequence homology of 32% and are phylogenetically distant. The (−)-enantiomer is known from plants including *Taiwania cryptomerioides* ([α]_D_^23^ = −102.9 (*c* 1.0, CHCl_3_)) [42], while the (+)-enantiomer has also been isolated from the liverwort *Scapania undulata* [43].

The (+)-T-muurolol synthase from *R. castenholzii* DSM 13941 contains the aspartate-rich motif (^81^DDQCD), the NSE triad (^221^NDVLSYPKE), the pyrophosphate sensor R-175 and the ^309^RY dimer. Closely related is the (+)-T-muurolol synthase from *Roseiflexus* sp. RS-1 [29] with 69% identical residues.

Heterologous expression of a third terpene synthase from *Streptosporangium roseum* DSM 43021 (accession number WP_043653400) and its incubation with FPP yielded the sesquiterpene alcohol **3**, identified as 4-*epi*-cubebol by GC–MS, and minor amounts of cubebol, germacrene D-4-ol and δ-cadinene (Figure S1, Supporting Information File 1), while GPP and GGPP did not yield any products. The ^13^C NMR spectrum and the ^13^C-DEPT135 spectrum of purified **3** showed no signals in the olefinic region, but one quarternary carbon (80.4 ppm) connected to a hydroxy function, four methyl groups (CH_3_), four methylene groups (CH_2_), five methine (CH) and one additional quarternary carbon, supporting a tricyclic structure (Table 2). The ^1^H,^1^H-COSY spectrum revealed three spin systems C2-3, C7-8-9-14, and C12-11-13 (Figure 2c) and the HMBC spectrum showed cross peaks between H-15 and C-3, C-4 and C-5, and between H-12/H-13 and C-11/C-7, giving evidence for the connectivities of the methyl groups. Further HMBC correlations between H-2 and C-1, C-3, C-5, C-6 and C-10 together with the highfield proton signals at 0.35 ppm (H-6) and 0.71 ppm (H-5) revealed a cyclopropane moiety. The relative configuration of **3** was determined by two-dimensional NOESY spectroscopy. Key correlations between H-15 and H-6, H-6 and H-8α (0.76 ppm), and H-8α with H-10 placed these hydrogens at the same face of the molecule, while NOE correlations between H-14 and H-5 and between H-5 and H-7 showed that these hydrogens are located at the opposite face. These data established the structure of 4-*epi*-cubebol (**3**). Its optical rotary power was determined as [α]_D_^24^ = +7.1 (*c* 0.29, CH_2_Cl_2_). This points to the same enantiomer as reported from the heartwood of *Cryptomeria japonica* [44], but the absolute configuration of **3** remains unknown.

**Table 2:** NMR data of 4-*epi*-cubebol (**3**) and 7-*epi*-α-eudesmol (**4**) in (^2^H_6_)benzene.

C^a^	**3** ^1^H (δ, m, *J*)^b^	^13^C (δ)^b^	**4** ^1^H (δ, m, *J*)^b^	^13^C (δ)^b^

1	–	35.1 (C_q_)	1.37 (m, 2H)	39.2 (CH_2_)
2	2.08 (dt, *J* = 11.8, *J* = 8.6, 1H) 1.39 (dt, *J* = 12.3, *J* = 8.2, 1H)	30.3 (CH_2_)	1.95 (m, 1H) 2.13 (m, 1H)	23.6 (CH_2_)
3	1.16 (ddd, *J* = 13.6, *J* = 11.2, *J* = 8.4, 1H) 1.46 (m, 1H)	36.9 (CH_2_)	5.36 (m, 1H)	121.3 (CH)
4	–	80.4 (C_q_)	–	135.8 (C_q_)
5	0.71(d, *J* = 3.0, 1H)	40.4 (CH)	2.24 (d, *J* = 13.2, 1H)	41.3 (CH)
6	0.35 (t, *J* = 3.0, 1H)	25.4 (CH)	1.27 (m, 1H) 1.86 (ddd, *J* = 13.7, *J* = 4.9, *J* = 4.8, 1H)	24.5 (CH_2_)
7	0.91 (m, 1H)	45.0 (CH)	1.54 (m, 1H)	42.7 (CH)
8	0.76 (m, 1H) 1.33 (m, 1H)	27.6 (CH_2_)	1.65 (m, 2H)	21.2 (CH_2_)
9	0.46 (dtd, *J* = 13.2, *J* = 11.8, *J* = 2.2, 1H) 1.53 (m, 1H)	32.2 (CH_2_)	1.28 (m, 1H) 1.48 (m, 1H)	38.0 (CH_2_)
10	1.69 (sept., *J* = 6.0, 1H)	30.7 (CH)	–	31.8 (C_q_)
11	1.48 (m, 1H)	34.1 (CH)	–	73.6 (C_q_)
12	0.94 (d, *J* = 6.7, 3H)	20.1 (CH_3_)	1.04 (s, 3H)	29.3 (CH_3_)
13	0.90 (d, *J* = 6.7, 3H)	20.3 (CH_3_)	1.05 (s, 3H)	28.6 (CH_3_)
14	0.99 (d, *J* = 6.5, 3H)	19.1 (CH_3_)	0.93 (s, 3H)	18.3 (CH_3_)
15	1.35 (s, 3H)	25.7 (CH_3_)	1.67 (m, 3H)	21.3 (CH_3_)

^a^Carbon numbering as in Figure 1. ^b^Chemical shifts δ in ppm; multiplicities: m = multiplet, s = singlet, d = doublet, sept = septet, br = broad; coupling constants *J* in Hz.

The (+)-4-*epi*-cubebol synthase from *S. roseum* DSM 43021 exhibits the aspartate-rich motif (^46^DDAFC), the NSE triad (^185^NDLISYAKE), the pyrophosphate sensor (R-139) and the ^271^RY dimer. The closest homolog with 97% identical residues is found in *S. roseum* NRRL B-2638 that likely also functions as (+)-4-*epi*-cubebol synthase. Furthermore, a 10-*epi*-cubebol synthase was recently identified from *Sorangium cellulosum* [45], but this enzyme exhibits only poor sequence identity (29%) to the *S. roseum* (+)-4-*epi*-cubebol synthase and must have evolved independently.

**Figure 3:**
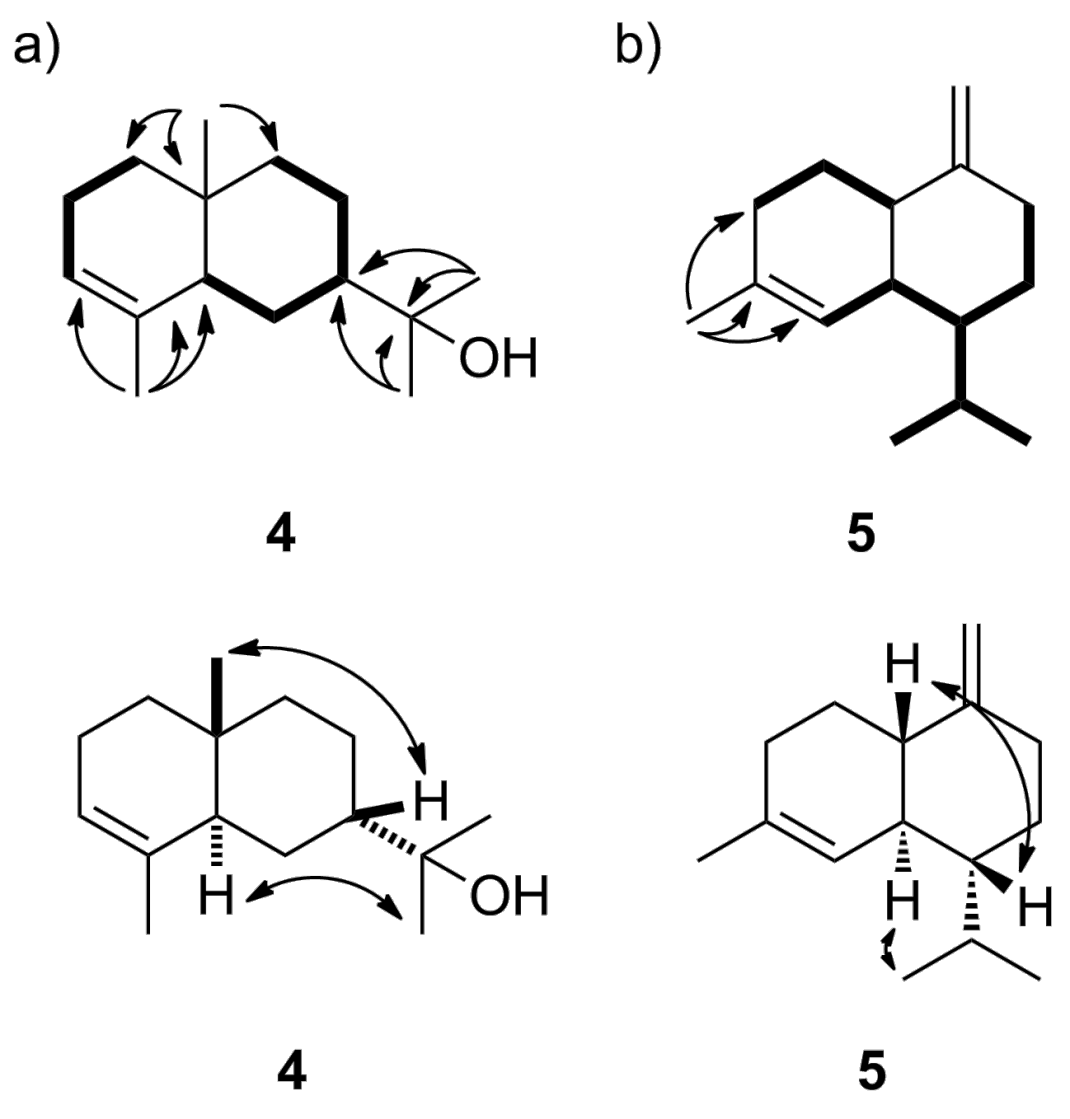
Contiguous spin systems observed by ^1^H,^1^H-COSY (bold), key HMBC and NOE correlations for a) 7-*epi*-α-eudesmol (**4**) and b) γ-cadinene (**5**).

Incubation of FPP with a terpene cyclase from *Streptomyces viridochromogenes* DSM 40736 (accession number WP_003994861) yielded a sesquiterpene alcohol **4**, that was identified as 7-*epi*-α-eudesmol by GC–MS [32], while GPP and GGPP were not accepted. Depending on the individual experiment, the conversion of FPP also yielded variable quantities of hedycaryol (Figure S1, Supporting Information File 1), the proposed biosynthetic intermediate towards **4** (vide infra). The ^13^C and the ^13^C-DEPT135 spectra of the purified compound showed signals for four methyl groups, five methylene groups, three methine and three quarternary carbons (Table 2). The ^1^H,^1^H-COSY spectrum revealed two contiguous spin systems C1-2-3 and C5-6-7-8-9 (Figure 3a). The location of the four methyl groups was deduced from HMBC cross peaks between H-15 and C-3, C-4 and C-5, between H-14 and C-1, C-5, C-9 and C-10, from H-12 to C-7, C-11 and C-13, and from H-13 to C-7, C-11 and C-12. Key signals in the NOESY spectrum were detected between H-7 and H-14 and from H-5 to H-12 which supported a *trans*-decalin system and the structure of 7-*epi*-α-eudesmol (**4**) for the enzyme product. Its optical rotary power was determined as [α]_D_^22^ = −51.3 (*c* 0.27, C_6_^2^H_6_). The opposite enantiomer was reported from *Eucalyptus macarthuri* ([α]_D_ = +30.5) [46], while **4** was also identified in several essential oils for example from *Hymenocrater longiflorus* or from *Juniperus oxycedrus* [47–48]. The absolute configuration of **4** has never been assigned.

The (−)-7-*epi*-α-eudesmol synthase from *S. viridochromogenes* DSM 40736 displays all highly conserved motifs including the aspartate-rich motif (^80^DDQFD), the NSE triad (^223^NDIHSFERE), the pyrophosphate sensor (R-177), and the ^317^RY dimer. Closely related enzymes are encoded in more than 35 of the genome sequenced streptomycetes with the enzyme from *S. chartreusis* NRRL 12338 as closest relative (94% identical sites).

Another terpene synthase from *Chitinophaga pinensis* DSM 2588 (accession number WP_012792334) converted FPP into a terpene hydrocarbon **5**, identified as γ-cadinene by GC–MS [32], besides traces of α- and δ-cadinene (Figure S1, Supporting Information File 1), while no reaction was observed with GPP or GGPP. The ^13^C and ^13^C-DEPT135 NMR spectra showed four signals in the olefinic region (one methylene, 103.4 ppm), one methine (122.5 ppm) and two quarternary carbons (152.7 ppm and 134.3 ppm), revealing the presence of one *exo*-methylene group and a second double bond in a ring. Furthermore, signals for three methyl groups, four methylene groups and four methine groups were observed in the spectra (Table 3). ^1^H,^1^H-COSY spectroscopy revealed two spin systems (C1-2-3 and C5-6-7(-11(-13)-12)-8-9), while key HMBC correlations between H-15 and C-3, C-4 and C-5 gave rise to the connectivity of the C-15 methyl group (Figure 3b). Strong NOE correlations between H-1 and H-7 and between H-6 and H-12 established the *trans*-decalin system. The optical rotary power was determined as [α]_D_^22^ = −32.3 (*c* 0.05, CH_3_OH) indicating the absolute configuration of (1*S*,6*S*,7*R*)-(−)-γ-cadinene which is the opposite enantiomer as reported from *Valeriana officinalis* ([α]_D_^20^ = +18.3 (*c* 0.16, CH_3_OH)) [49].

**Table 3:** NMR data of γ-cadinene (**5**) and (*E*)-β-caryophyllene (**6**) in (^2^H_6_)benzene.

C^a^	**5** ^1^H (δ, m, *J*)^b^	^13^C (δ)^b^	**6** ^1^H (δ, m, *J*)^b^	^13^C (δ)^b^

1	1.76 (m, 1H)	44.6 (CH)	1.70 (m, 1H) 2.36 (m, 1H)	40.4 (CH_2_)
2	1.53 (m, 1H) 1.93 (m, 1H)	26.1 (CH_2_)	2.29 (q, *J* = 9.1, 1H)	48.9 (CH)
3	1.87 (m, 1H) 1.92 (m, 1H)	30.9 (CH_2_)	–	154.6 (C_q_)
4	–	134.6 (C_q_)	1.93 (m, 1H) 2.13 (m, 1H)	35.1 (CH_2_)
5	5.64 (s, 1H)	122.9 (CH)	1.94 (m, 1H) 2.31 (m, 1H)	28.8 (CH_2_)
6	1.75 (m, 1H)	45.6 (CH)	5.36 (dd, *J* = 9.1, *J* = 6.1, 1H)	124.9 (CH)
7	1.19 (m, 1H)	47.3 (CH)	–	135.3 (C_q_)
8	1.13 (dq, *J* = 4.2, *J* = 12.4, 1H) 1.67 (m, 1H)	26.8 (CH_2_)	1.88 (m, 1H) 2.00 (m, 1H)	40.4 (CH_2_)
9	2.38 (ddd, *J* = 13.1, *J* = 3.9, *J* = 2.9, 1H) 2.00 (m, *J* = 13.2, *J* = 4.7, 1H)	36.8 (CH_2_)	1.31 (m, 1H) 1.37 (m, 1H)	29.7 (CH_2_)
10	–	153.1 (C_q_)	1.67 (m, 1H)	53.8 (CH)
11	2.16 (dqq, *J* = 3.2, *J* = 7.0, *J* = 7.1, 1H)	26.5 (CH)	–	33.2 (C_q_)
12	0.88 (d, *J* = 7.1, 3H)	21.7 (CH_3_)	1.00 (s, 3H)	30.2 (CH_3_)
13	0.73 (d, *J* = 6.9, 3H)	15.3 (CH_3_)	0.94 (s, 3H)	22.8 (CH_3_)
14	4.82 (s, 1H) 4.70 (s, 1H)	103.8 (CH_2_)	1.56 (s, 3H)	16.4 (CH_3_)
15	1.65 (s, 3H)	24.1 (CH_3_)	4.87 (s, 1H) 5.03 (s, 1H)	112.2 (CH_2_)

^a^Carbon numbering as in Figure 1. ^b^Chemical shifts δ in ppm; multiplicities: m = multiplet, s = singlet, d = doublet, sept = septet, br = broad; coupling constants *J* in Hz.

The (−)-γ-cadinene synthase from *C. pinensis* exhibits all highly conserved motifs including the aspartate-rich motif (^82^DDQCD), the NSE triad (^220^NDIFSCAKE), the pyrophosphate sensor (R-174) and the ^309^RY dimer. No closely related enzymes are encoded in other bacteria, the closest homolog being an unidentified protein from *Shimazuella kribbensis* DSM 45090 (accession number WP_028777381, 30% identity).

Finally a terpene synthase from *Saccharothrix espanaensis* DSM 44229 (accession number WP_015102836) was incubated with FPP to yield a sesquiterpene hydrocarbon as single product (Figure S1, Supporting Information File 1) whose NMR data (Table 3) matched those reported for (*E*)-β-caryophyllene (**6**) [50]. The optical rotary power was determined as [α]_D_^24^ = −14.0 (*c* 0.13, CH_2_Cl_2_), pointing to the same absolute configuration as of synthetic (2*S*,10*R*)-(−)-(*E*)-β-caryophyllene ([α]_D_^20^ = −13.0 (*c* 1.5, CHCl_3_) [50]). The compound is exceptionally widespread in nature and was, e.g., reported from the essential oils from *Syzygium aromaticum* [51], *Cannabis sativa* [52] and *Rosmarinus officinalis* [53].

The *S. espanaensis* (−)-(*E*)-β-caryophyllene synthase contains the aspartate-rich motif (^85^DDQFD), the NSE triad (^225^NDVASTIKE), the pyrophosphate sensor (R-179) and an altered RY dimer (^320^RF). No closely related (−)-(*E*)-β-caryophyllene synthase is encoded in other sequenced bacteria, but the enzyme from *S. espanaensis* shows highest identity with pentalenene synthases (39% identical residues with pentalenene synthase from *Streptomyces exfoliatus* UC5319) [11]. Intriguingly, both compounds are made from FPP via an initial 1,11-cyclisation.

**Scheme 1:**
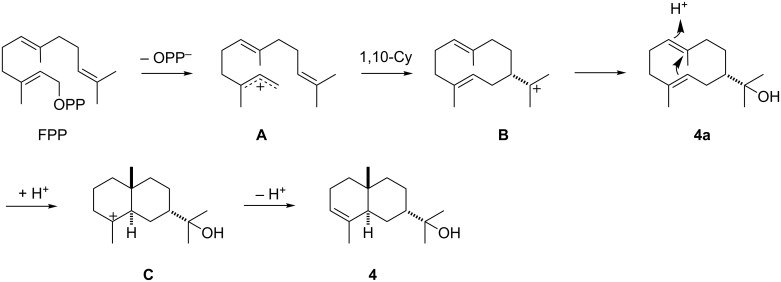
Biosynthetic pathway from FPP to 7-*epi*-α-eudesmol (**4**).

The cyclisation mechanisms of the bacterial terpene cyclases were investigated by isotopic labelling experiments. The proposed biosynthesis of 7-*epi*-α-eudesmol (**4**) starts with a 1,10-cyclisation of FPP to the (*E*,*E*)-germacradienyl cation (**B**) which is attacked by water to form hedycaryol (**4a**). Its reprotonation at C-1 initiates a second cyclisation to cation **C** that undergoes deprotonation to **4** (Scheme 1).

**Figure 4:**
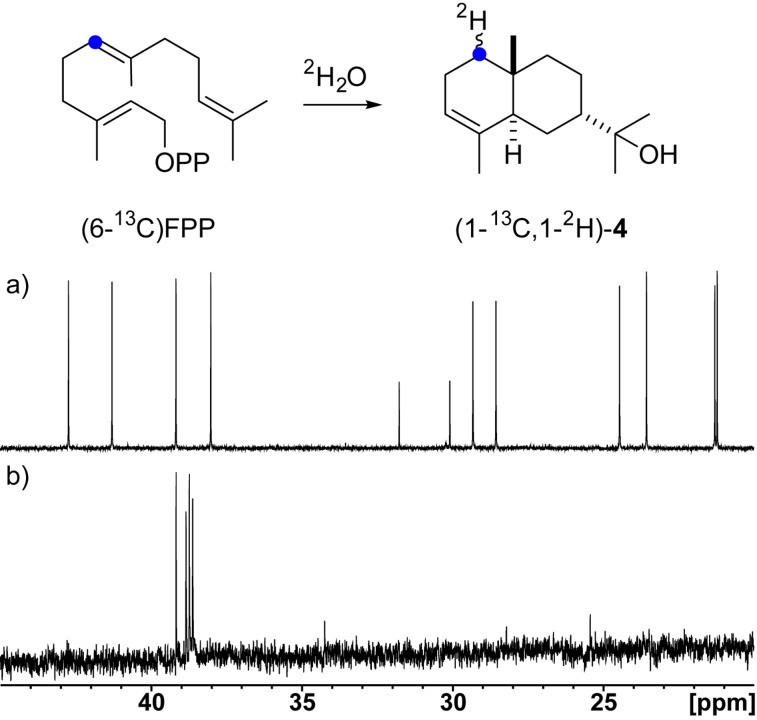
Incubation experiments with (6-^13^C)FPP and the 7-*epi*-α-eudesmol synthase in deuterium oxide. a) ^13^C NMR spectrum of unlabelled **4** and b) ^13^C NMR spectrum of the extract from the incubation experiment with (6-^13^C)FPP in ^2^H_2_O.

This biosynthetic model was tested by incubation of (6-^13^C)FPP with the 7-*epi*-α-eudesmol synthase in deuterium oxide to follow the reprotonation of the neutral intermediate **4a** [54–55]. A simple extraction of the reaction mixture with (^2^H_6_)benzene and direct ^13^C NMR analysis resulted in a highfield shifted triplet at 38.7 ppm (Δδ = −0.5 ppm, ^1^*J*_C,D_ = 19.4 Hz), demonstrating the introduction of deuterium at C-6 of **4** (Figure 4). The singlet at 39.2 ppm is observed due to residual water in the enzyme reaction.

Germacranes have been suggested as general precursors for eudesmanes [56]. Their interconversion could be a non-enzymatic process catalysed by weak acids, e.g., during work-up, but the acid-catalysed conversion of **4a** is known to give a mixture of α-, β-, and γ-eudesmol [57], but not 7-*epi*-α-eudesmol, which demonstrates the participation of the enzyme to fix **4a** in the correct conformation for its cyclisation to **4**. The detection of **4a** in the extracts from enzyme incubations of FPP (Figure S1, Supporting Information File 1) strongly supports its role as a neutral intermediate towards **4**.

**Figure 5:**
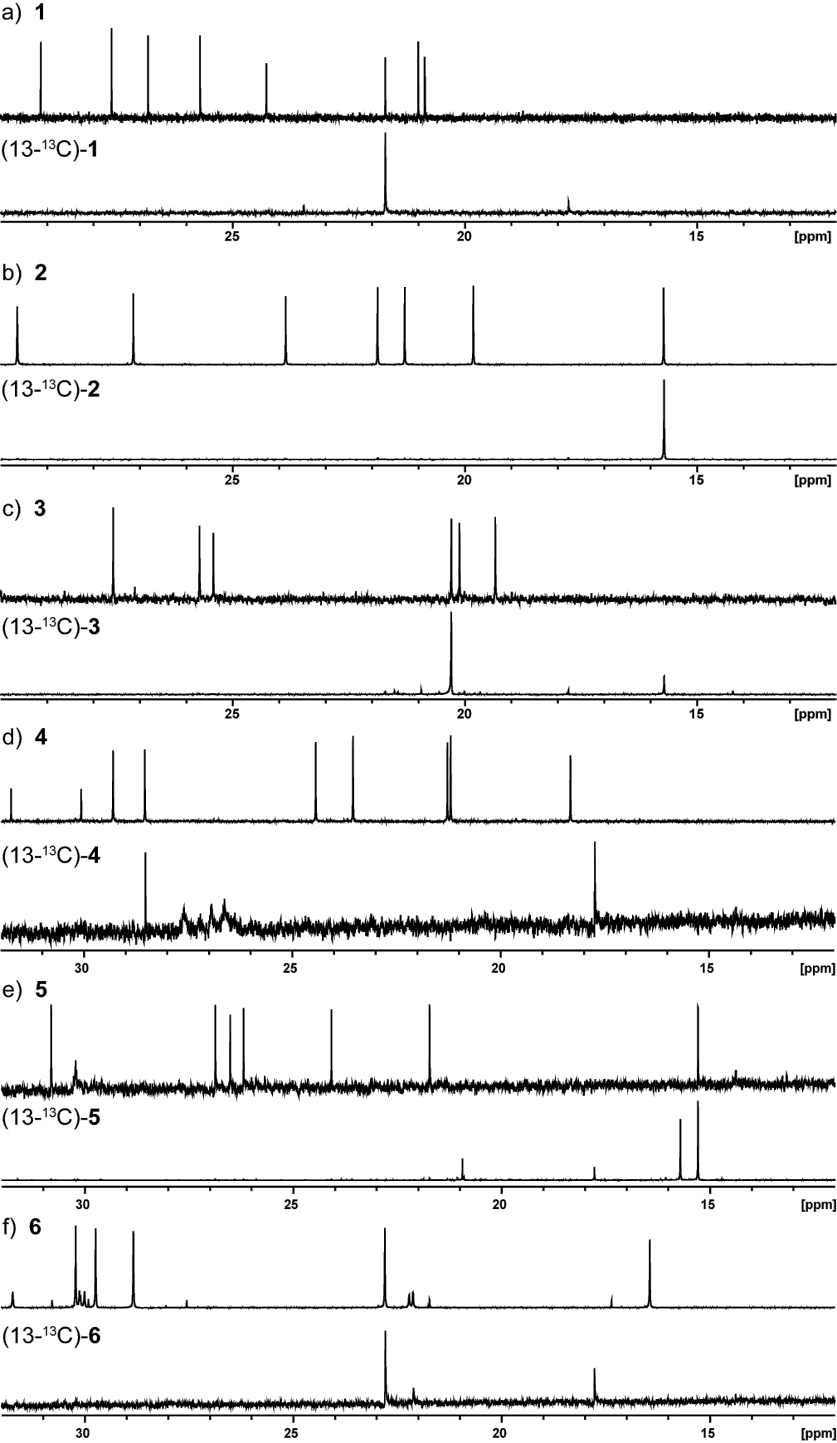
Incubation experiments with (13-^13^C)FPP. ^13^C NMR spectra of a) unlabelled **1** and (13-^13^C)-**1**, b) unlabelled **2** and (13-^13^C)-**2**, c) unlabelled **3** and (13-^13^C)-**3**, d) unlabelled **4** and (13-^13^C)-**4**, e) unlabelled **5** and (13-^13^C)-**5**, f) unlabelled **6** and (13-^13^C)-**6**.

The stereochemical courses of the terpene cyclisations in terms of the fate of the terminal *E*- and *Z*-methyl groups of FPP (C-12 and C-13) during the formation of the products **1** to **6** were investigated by incubation experiments with stereospecifically labelled (13-^13^C)FPP. After enzymatic conversion and extraction with (^2^H_6_)benzene the ^13^C NMR spectra were directly recorded, revealing one strongly enhanced signal for the methyl group originating from C-13 of FPP in each case. For (−)-α-amorphene synthase a signal was observed at 21.7 ppm, but not at 21.0 ppm (Figure 5a). The incubation experiment with the (+)-T-muurolol synthase and (13-^13^C)FPP resulted in a strong signal at 15.3 ppm, but not at 21.7 ppm (Figure 5b), whereas the incubation experiment with the (+)-4-*epi*-cubebol synthase gave a signal at 20.3 ppm, but not at 20.1 ppm (Figure 5c). Enzymatic conversion of (13-^13^C)FPP with (−)-7-*epi*-α-eudesmol synthase yielded an enhanced signal at 28.6 ppm, but no signal at 29.3 ppm (Figure 5d), while for (−)-γ-cadinene synthase a signal was detected at 15.3 ppm, but not at 21.7 ppm (Figure 5e). Finally, (13-^13^C)FPP with (−)-(*E*)-β-caryophyllene synthase resulted in a signal at 22.8 ppm, but not at 30.2 ppm (Figure 5f). These experiments demonstrate that in case of all six sesquiterpene cyclases the substrate FPP is converted with a strict stereochemical course with respect to the fate of the diastereotopic methyl groups in FPP. These findings are in agreement with those reported for several other terpene cyclases [58–60], while the (1*R*,4*R*,5*S*)-guaia-6,10(14)-diene synthase from *Fusarium fujikuroi* shows a relaxed stereochemical course in this aspect [61].

## Conclusion

We have isolated and characterized the enzyme products of six bacterial terpene synthases by extensive one and two-dimensional NMR spectroscopic analysis and determination of the optical rotary powers. In total, two terpene synthases from *Streptomyces viridochromogenes* ((−)-α-amorphene (**1**) and (−)-7-*epi*-α-eudesmol synthase (**4**)), the (+)-T-muurolol synthase (**2**) from *Roseiflexus castenholzii*, the (+)-4-*epi*-cubebol synthase (**3**) from *Streptosporangium roseum*, the (−)-γ-cadinene synthase (**5**) from *Chitinophaga pinensis* and the (−)-(*E*)-β-caryophyllene synthase (**6**) from *Saccharothrix espanaensis* were identified. The identified main and side products were the same as detected in headspace extracts from *E. coli* during heterologous expression of the terpene cyclase genes [32–33], confirming that the in vitro and in vivo experiments give the same results. Additionally we have demonstrated that the 7-*epi*-α-eudesmol biosynthesis proceeds via reprotonation of the neutral intermediate hedycaryol by usage of (6-^13^C)FPP as substrate in an incubation experiment with recombinant purified enzyme in deuterium oxide. Finally, incubation experiments with (13-^13^C)FPP and all six purified recombinant terpene synthases were performed to investigate the stereochemical course of the biosynthesis of the terminal *E*- and *Z*-methyl groups and to locate this diagnostic ^13^C marker by NMR spectroscopy in the obtained products. All six enzymes showed a strict stereochemical course for the FPP cyclisation, as is reported for most, but not all other terpene cyclases that were investigated for this aspect. It is interesting to note that the bacterial sesquiterpenes identified in this study are in most cases the opposite enantiomers as known from plants, demonstrating that bacterial enzymes are worth to be investigated to make these opposite enantiomers accessible. It is also tempting to speculate that the optical antipodes used by bacteria and plants represent two chemical communicatory channels that may not interfer, even if the same habitat is occupied by the prokaryotic and eukaryotic terpene producing organisms.

## Experimental

### NMR and IR spectroscopic analysis

NMR spectra of isolated natural products were recorded on a Bruker AV Avance DMX-500 (500 MHz), a Bruker AV III HD Cryo (700 MHz) and a Bruker AV III HD Prodegy (500 MHz) spectrometer, and were referenced against solvent signals (^1^H NMR: (^2^H_6_)benzene δ = 7.16 ppm, ^13^C NMR: (^2^H_6_)benzene δ = 128.06 ppm. IR spectra of the isolated natural products were recorded on a Bruker alpha FTIR spectrometer.

### GC–MS and GC–MS–QTOF analysis

GC–MS analyses were carried out with a 7890B gas chromatograph connected to a 5977A inert mass detector (Agilent) fitted with a HP5-MS fused silica capillary column (30 m, 0.25 mm i. d., 0.50 μm film). Instrumental parameters were (1) inlet pressure, 77.1 kPa, He 23.3 mL min^−1^, (2) injection volume, 2 μL, (3) transfer line, 250 °C, and (4) electron energy 70 eV. The GC was programmed as follows: 5 min at 50 °C increasing at 5 °C min^−1^ to 320 °C, and operated in split mode (10:1, 60 s valve time). The carrier gas was He at 1 mL min^−1^. Retention indices (*I*) were determined from a homologous series of *n*-alkanes (C_7_−C_40_). Compound identification of the side products of terpene cyclases was based on a matching mass spectrum to a library mass spectrum and retention index to published data.

HRMS analyses were carried out with a 7890B gas chromatograph connected to a 7200 accurate-mass Q-TOF mass detector (Agilent) eqipped with a HP5-MS fused silica capillary column (30 m, 0.25 mm i. d., 0.50 μm film). Instrumental parameters were (1) inlet pressure, 83.2 kPa, He 24.6 mL min^−1^, (2) injection volume, 2 μL, (3) transfer line, 250 °C, and (4) electron energy 70 eV. The GC was programmed for HRMS as follows: 5 min at 50 °C increasing at 5 °C min^−1^ to 320 °C, and operated in split mode (50:1, 60 s valve time). The carrier gas was He at 1 mL min^−1^.

### Incubation experiments with the natural substrate and product isolation

*E. coli* BL 21 transformants, including plasmids carrying the terpene synthase gene (Table 3), were inoculated in a 2YT liquid preculture (tryptone 16 g, yeast extract 10 g, NaCl 5 g, water 1 L) containing kanamycin (50 mg/L) overnight. The *E. coli* BL 21 preculture was used to inoculate an expression culture of larger volume (for volumes cf. Table 3) containing kanamycin (50 mg/L). The cells were grown to an OD_600_ = 0.4 at 37 °C and 160 rpm. After cooling of the culture to 18 °C for 30 minutes, IPTG (0.4 mM) was added. For expression the culture was incubated at 18 °C and 160 rpm overnight. *E. coli* cells were harvested by centrifugation at 4 °C and 3600 rpm for 60 min. The pellets were resuspended in binding buffer (for each 1 L culture 15 mL of buffer were used; 20 mM Na_2_HPO_4_, 0.5 M NaCl, 20 mM imidazole, 1 mM MgCl_2_, pH 7.0). The disruption of the cells was done by ultra-sonication on ice for 8 × 60 s for each portion of cells from 2 L of culture. The soluble enzymes were harvested at 4 °C and 11000 rpm by centrifugation (2 × 10 min). Protein purification was performed by Ni^2+^-NTA affinity chromatography with Ni^2+^-NTA superflow (Novagen) using binding buffer and elution buffer (20 mM Na_2_HPO_4_, 0.5 M NaCl, 0.5 M imidazole, 1 mM MgCl_2_, pH 7.0). Incubation experiments were performed with the pure protein fractions and the natural substrate FPP (amount and final concentration of FPP see Table 3) at 28 °C overnight. The incubation experiment of FPP with the purified γ-cadinene synthase was performed with a syringe pump, which added the substrate FPP over 1 h to the enzyme fraction. The reaction mixture was extracted with 3 × 50–80 mL hexane. The combined organic layers were dried with MgSO_4_ and concentrated under reduced pressure. Column chromatography on silica gel of the crude product with pentane/diethyl ether yielded the pure sesquiterpene (amount see Table 4) for structure elucidation by NMR and for determination of the optical rotary power.

**Table 4:** Yields from enzyme incubation experiments.

terpene synthase	culture^a^	amount of FPP, concentration	product

(−)-α-amorphene (**1**)	4 L	60 mg, 0.5 mg/mL	6 mg
(+)-T-muurolol (**2**)	8 L	120 mg; 0.8 mg/mL	32 mg
(+)-4-*epi*-cubebol (**3**)	4 L	50 mg; 0.5 mg/mL	7 mg
(−)-7-*epi*-α-eudesmol (**4**)	4 L	48 mg; 0.3 mg/mL	6 mg
(−)-γ-cadinene (**5**)	6 L	60 mg; 0.3 mg/mL	2.6 mg
(−)-(*E*)-β-caryophyllene (**6**)	8 L	28 mg; 0.2 mg/mL (syringe pump)	2.1 mg

^a^Enzyme preparation was made from the indicated culture volume of the *E. coli* expression strain.

#### Spectroscopic data of isolated terpenes

**(−)-α-Amorphene (1):** GC (HP 5): *I* = 1482 (literature (HP 5): *I* = 1483 [62]); MS (EI, 70 eV) *m*/*z* (%): 204 (31), 189 (12), 175 (5), 161 (57), 147 (12), 133 (18), 119 (34), 105 (100), 94 (67), 79 (25), 69 (11), 55 (14), 41 (25); HRMS (TOF) *m*/*z*: [M]^+^ calcd for C_15_H_24_^+^, 204.1873; found, 204.1872; IR (diamond ATR) 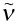: 2964 (m), 2925 (m), 2873 (m), 1670 (w), 1447 (w), 1412 (w), 1377 (w), 1259 (s), 1090 (s), 1016 (s), 866 (w), 797 (s), 700 (w), 662 (w) cm^−1^.

**(+)-T-Muurolol (2):** GC (HP 5): *I* = 1640 (literature (HP 5): *I* = 1640 [62]); MS (EI, 70 eV) *m*/*z* (%): 222 (5), 204 (54), 189 (9), 179 (6), 161 (64), 149 (9), 133 (11), 121 (70), 105 (44), 95 (100), 79 (35), 71 (30), 59 (19), 43 (67); HRMS (TOF) *m*/*z*: ([M]^+^ calcd for C_15_H_26_O^+^, 222.1978; found, 222.1971; IR (diamond ATR) 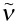: 3357 (br, m), 3009 (w), 2956 (m), 2931 (m), 2897 (m), 2869 (m), 2830(w), 1450 (m), 1368 (m), 1298 (w), 1232 (w), 1192 (m), 1143 (m), 1036 (m), 1016 (w), 933 (w), 910 (m), 898 (m), 831 (w) cm^−1^.

**(+)-4-*epi*-Cubebol (3):** GC (HP 5): *I* = 1495 (literature (HP 5): *I* = 1493 [62]); MS (EI, 70 eV) *m*/*z* (%): 222 (3), 207 (53), 189 (6), 179 (9), 161 (100), 147 (8), 133 (12), 119 (40), 105 (58), 91 (34), 81 (30), 69 (12), 55 (18), 43 (49); HRMS (TOF) *m*/*z*: [M]^+^ calcd for C_15_H_26_O^+^, 222.1978; found, 222.1982; IR (diamond ATR) 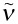: 3353 (br, m), 2956 (m), 2925 (m), 2869 (m), 1722 (m), 1639 (w), 1446 (m), 1376 (s), 1319 (m), 1258 (w), 1180 (m), 1081 (s), 955 (m), 910 (w), 837 (m), 802 (w) 614 (w), 539 (w) cm^−1^.

**(−)-7-*epi*-α-Eudesmol (4):** GC (HP 5): *I* = 1661 (literature (HP 5): *I* = 1662 [62]); MS (EI, 70 eV) *m*/*z* (%): 222 (1), 204 (23), 189 (15), 175 (5), 161 (100), 143 (10), 133 (13), 122 (65), 107 (38), 93 (20), 81 (22), 67 (12), 59 (24), 41 (13); HRMS (TOF) *m*/*z*: [M − H_2_O]^+^ calcd for C_15_H_26_O^+^, 204.1873, found. 204.1870; IR (diamond ATR) 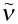: 3388 (br, m), 2966 (s), 2910 (s), 2850 (s), 1662 (w), 1440 (s), 1376 (w), 1281 (m), 1260 (m), 1220 (s), 1139 (m), 1101 (m), 1021 (s), 937 (w), 873 (m), 841 (s) 798 (w), 755 (w), 707 (w), 649 (w), 568 (w) cm^−1^.

**(−)-γ-Cadinene (5):** GC (HP 5): *I* = 1514 (literature (HP 5): *I* = 1513 [62]); MS (EI, 70 eV) *m*/*z* (%): 204 (49), 189 (9), 176 (5), 161 (100), 148 (11), 133 (31), 119 (40), 105 (48), 91 (31), 75 (19), 67 (10), 55 (9), 41(11); HRMS (TOF) *m*/*z*: [M]^+^ calcd for C_15_H_24_^+^, 204.1873; found, 204.1865; IR (diamond ATR) 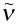: 2925 (s), 2850 (s), 1525 (m), 1458 (s), 1366 (s), 1302 (s), 1192 (w), 1087 (s), 1030 (s), 963 (m), 921 (w), 823 (s), 691 (m) 608 (s) cm^−1^.

**(−)-(*E*)-β-Caryophyllene (6):** GC (HP 5): *I* = 1429 (literature (HP 5): *I* = 1428 [63]); MS (EI, 70 eV) *m*/*z* (%): 204 (10), 189 (23), 175 (12), 161 (41), 147 (34), 133 (100), 120 (46), 105 (59), 93 (96), 79 (60), 69 (67), 55 (25), 41 (45); HRMS (TOF) *m*/*z*: [M]^+^ calcd for C_15_H_24_^+^, 204.1873; found, 204.1868; IR (diamond ATR) 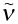: 3066 (w), 2924 (s), 2856 (s), 1670 (m), 1631 (m), 1449 (s), 1382 (s), 1367 (s), 1276 (m), 1257 (w), 1227 (w), 1182 (m), 1106 (w), 1067 (w); 1019 (w), 936 (m), 885 (s), 842 (m), 813 (m), 764 (w) 742 (s), 641 (w), 544 (m) cm^−1^.

#### Incubation experiments with (^13^C)FPPs

For each incubation experiment a 0.5 L 2YT liquid culture (containing kanamycin (50 mg/L)) of *E. coli* BL 21 transformants was inoculated from an overnight preculture. The cells were grown to an OD_600_ = 0.4 before IPTG (0.4 mM) was added. The cultures were incubated at 18 °C and 160 rpm overnight. *E. coli* cells were harvested by centrifugation at 4 °C and 8000 rpm for 10 min. Protein purification was performed by Ni^2+^-NTA affinity chromatography with Ni^2+^-NTA superflow (Novagen) as reported above. For the incubation experiment in ^2^H_2_O with 7-*epi*-α-eudesmol synthase and (6-^13^C)FPP, the soluble enzyme fraction was washed with binding buffer (20 mM Na_2_HPO_4_, 0.5 M NaCl, 20 mM imidazole, 1 mM MgCl_2_, pH 7.0 in ^2^H_2_O) and then eluted twice with 10 mL elution buffer (20 mM Na_2_HPO_4_, 0.5 M NaCl, 0.5 M imidazole, 1 mM MgCl_2_, pH 7.0 in ^2^H_2_O). Each pure protein fraction from 0.5 L 2YT liquid culture was concentrated with a Vivaspin20 concentration tube (MWCO 30000, Sartorius Stedim, Göttingen) for 0.5 to 1.5 h at 6000 rpm to 2 mL enzyme fraction. Incubation experiments were performed with the pure protein (2 mL) and the ^13^C-labeled substrate (0.8 mg to 3.0 mg in 2 mL ^2^H_2_O or H_2_O) at 28 °C overnight. The reaction mixture was extracted with 2 × 0.4 mL (^2^H_6_)benzene and directly measured by NMR.

## Supporting Information

File 1 Gas chromatograms of extracts from enzyme reactions and NMR spectra of compounds **1**–**5**.
