# Supplementary material for: Mechanistic investigations on six bacterial terpene cyclases
Source: Beilstein J Org Chem. 2016 Aug 15;12:1839–50. doi: 10.3762/bjoc.12.173 (PMC5082573; doi:10.3762/bjoc.12.173)
Supplement: File 1 — Gas chromatograms of extracts from enzyme reactions and NMR spectra of compounds 1–5. [file Beilstein_J_Org_Chem-12-1839-s001.pdf]

Supporting Information

for

Mechanistic investigations on six bacterial terpene cyclases

Patrick Rabe, Thomas Schmitz and Jeroen S. Dickschat*

Address: Kekulé-Institute of Organic Chemistry and Biochemistry, University of Bonn, Gerhard-Domagk-Straße 1, 53121 Bonn, Germany

Email: Jeroen S. Dickschat - dickschat@uni-bonn.de

* Corresponding author

Gas chromatograms of extracts from enzyme reactions and NMR spectra of compounds 1–5

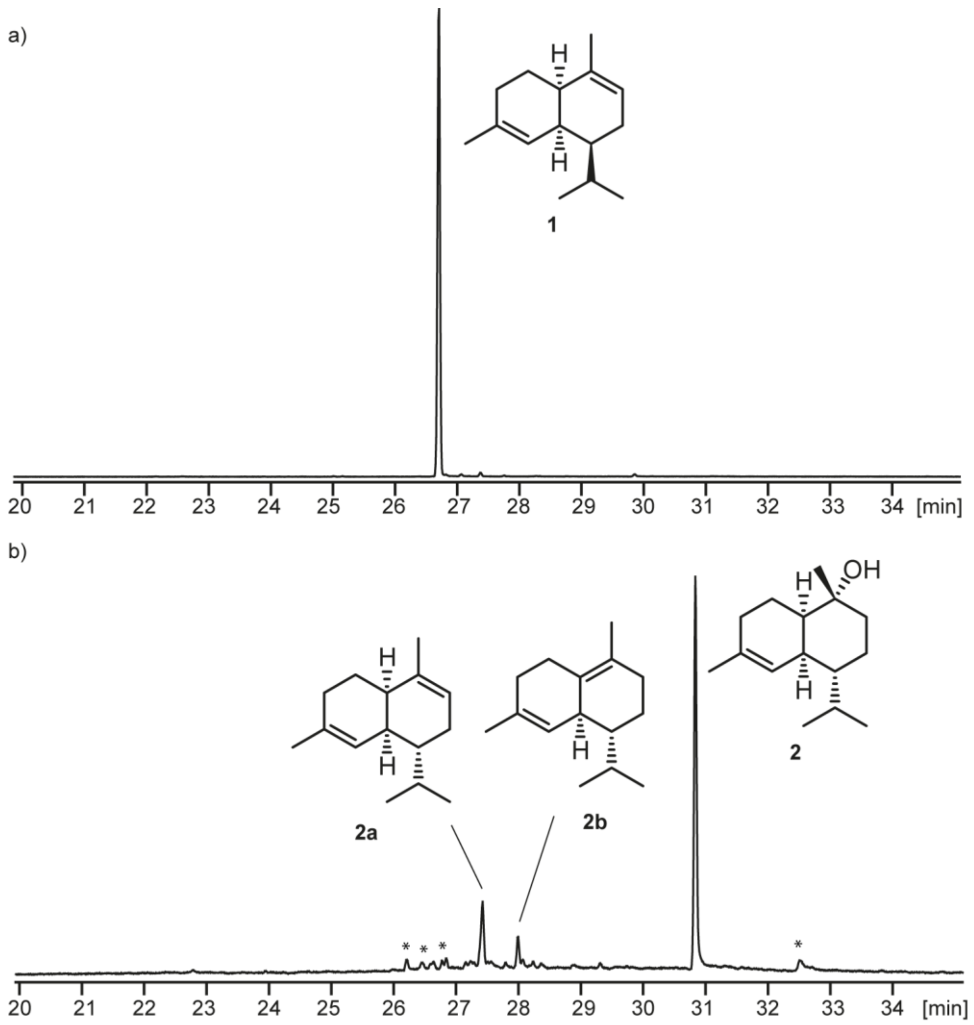

Figure S1: Total ion chromatograms of extracts from incubation experiments of FPP with a) (–)-α-amorphene synthase from *S. viridochromogenes* and b) (–)-7-*epi*-α-eudesmol synthase from *S. viridochromogenes*. Asterisks indicate unidentified compounds and contaminants such as plasticisers.

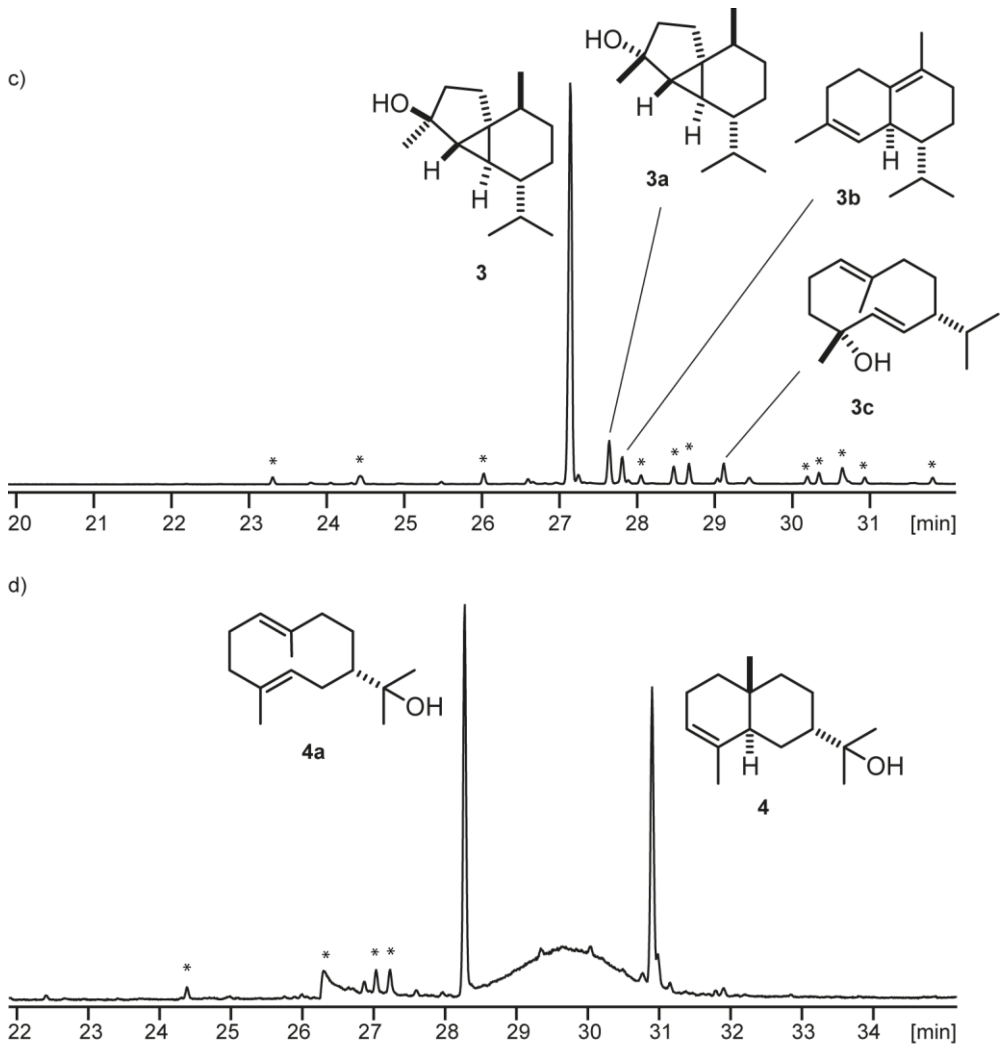

Figure S1 (continued): Total ion chromatograms of extracts from incubation experiments of FPP with c) (+)-T-muurolol synthase from *Roseiflexus castenholzii* and d) (+)-4-*epi*-cubebol synthase from *Streptosporangium roseum*. Asterisks indicate unidentified compounds and contaminants such as plasticisers.

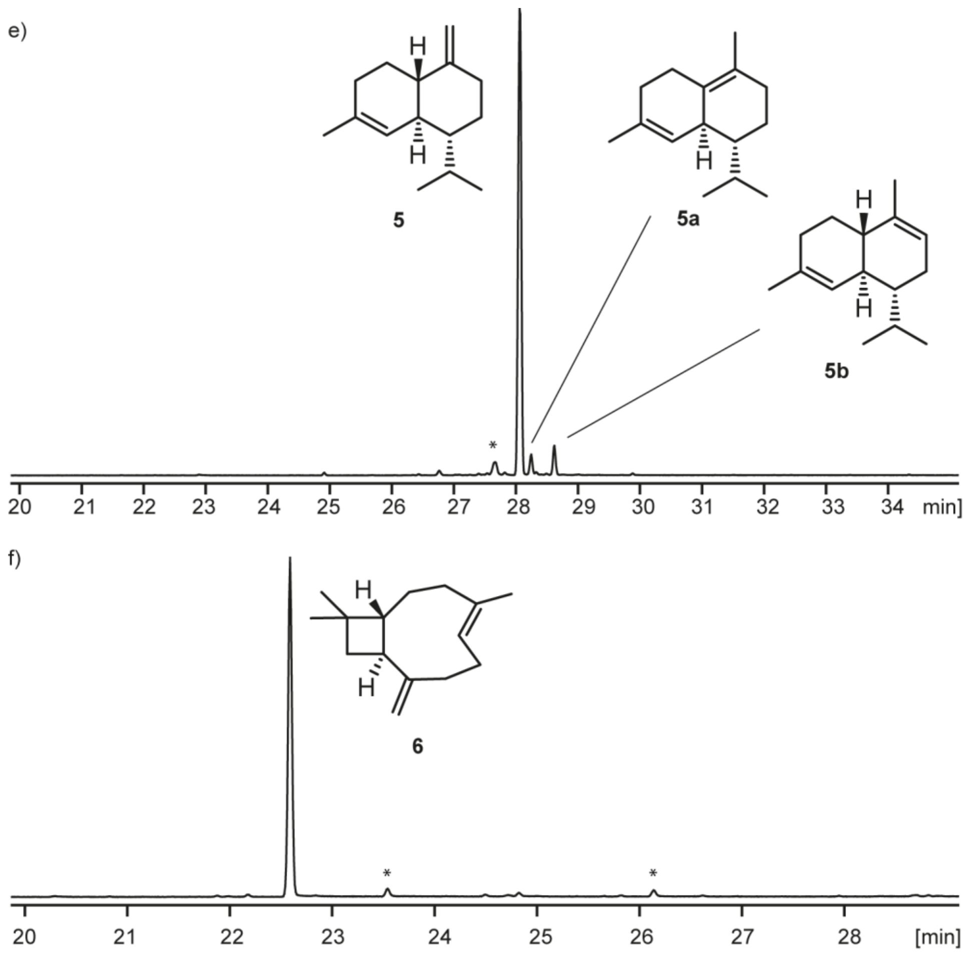

Figure S1 (continued): Total ion chromatograms of extracts from incubation experiments of FPP with e) (–)-γ-cadinene synthase from *Chitinophaga pinensis* and f) (–)-(*E*)-β-caryophyllene synthase from *Saccharothrix espanaensis*. Asterisks indicate unidentified compounds and contaminants such as plasticisers.

Table S1: Terpenes identified in hexane extracts of incubation experiments with FPP and recombinant purified terpene cyclases

**Compound**[a]	***I***[b]	***I* (Lit.)**[c]
α-muurolene (**2a**)	1504	1500
cubebol (**3a**)	1517	1514
δ-cadinene (**2b**, **3b**, **5a**)	1523	1520
α-cadinene (**5b**)	1546	1539
hedycaryol (**4a**)	1549	1546
germacrene D-4-ol (**3c**)	1577	1574

[a] Compound numbers refer to numbers in Figure 1. [b] Measured retention index on a HP5-MS column (data for main compounds **1** – **6** are given in the main text). [c] Retention index on a HP5-MS column from: Adams, R. P. *Identification of Essential Oil Components by Gas Chromatography/ Mass Spectrometry*, Allured, Carol Stream, **2009**.

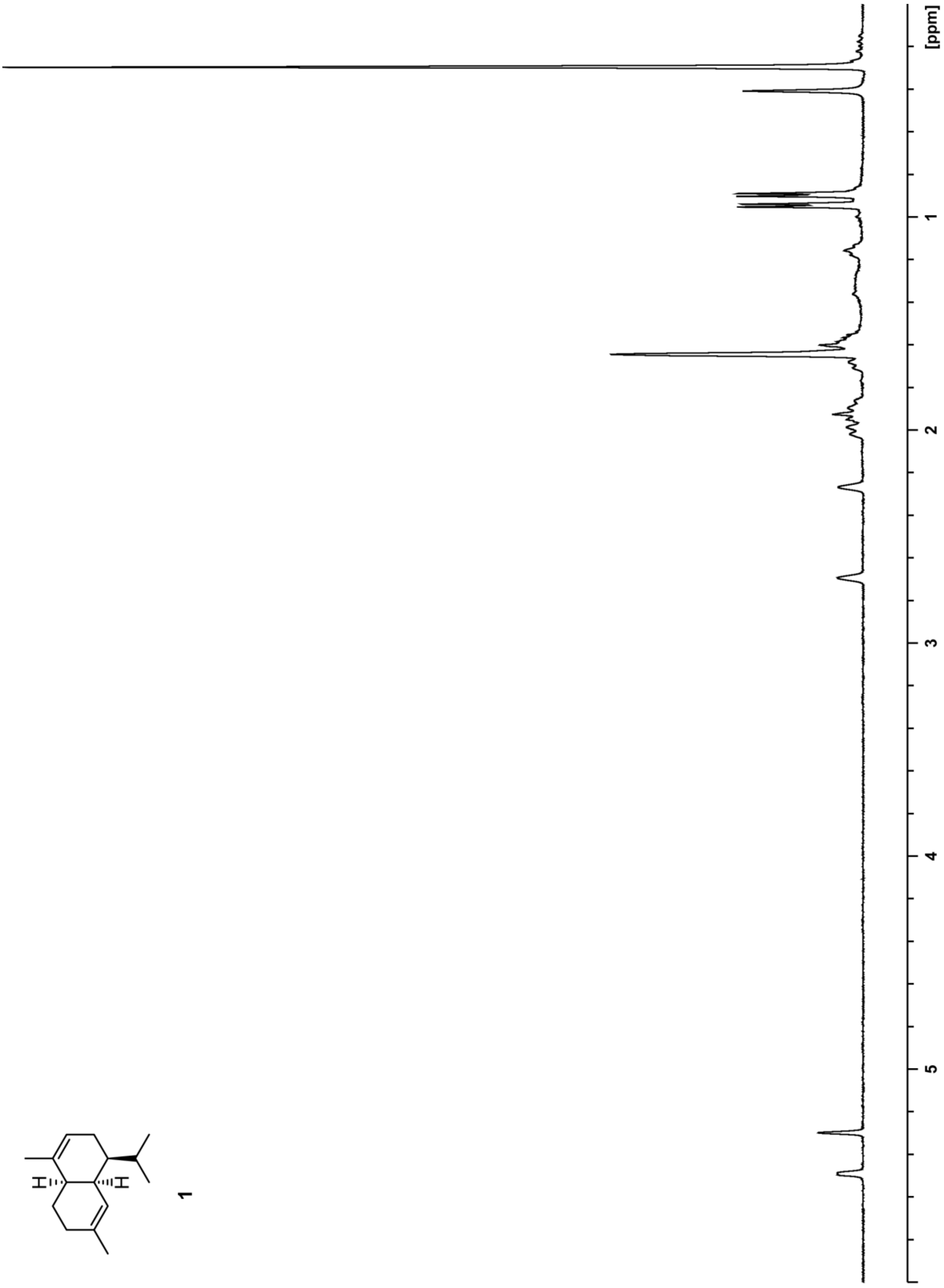

Figure S2: ^{1}H NMR spectrum of **1** at 500 MHz.

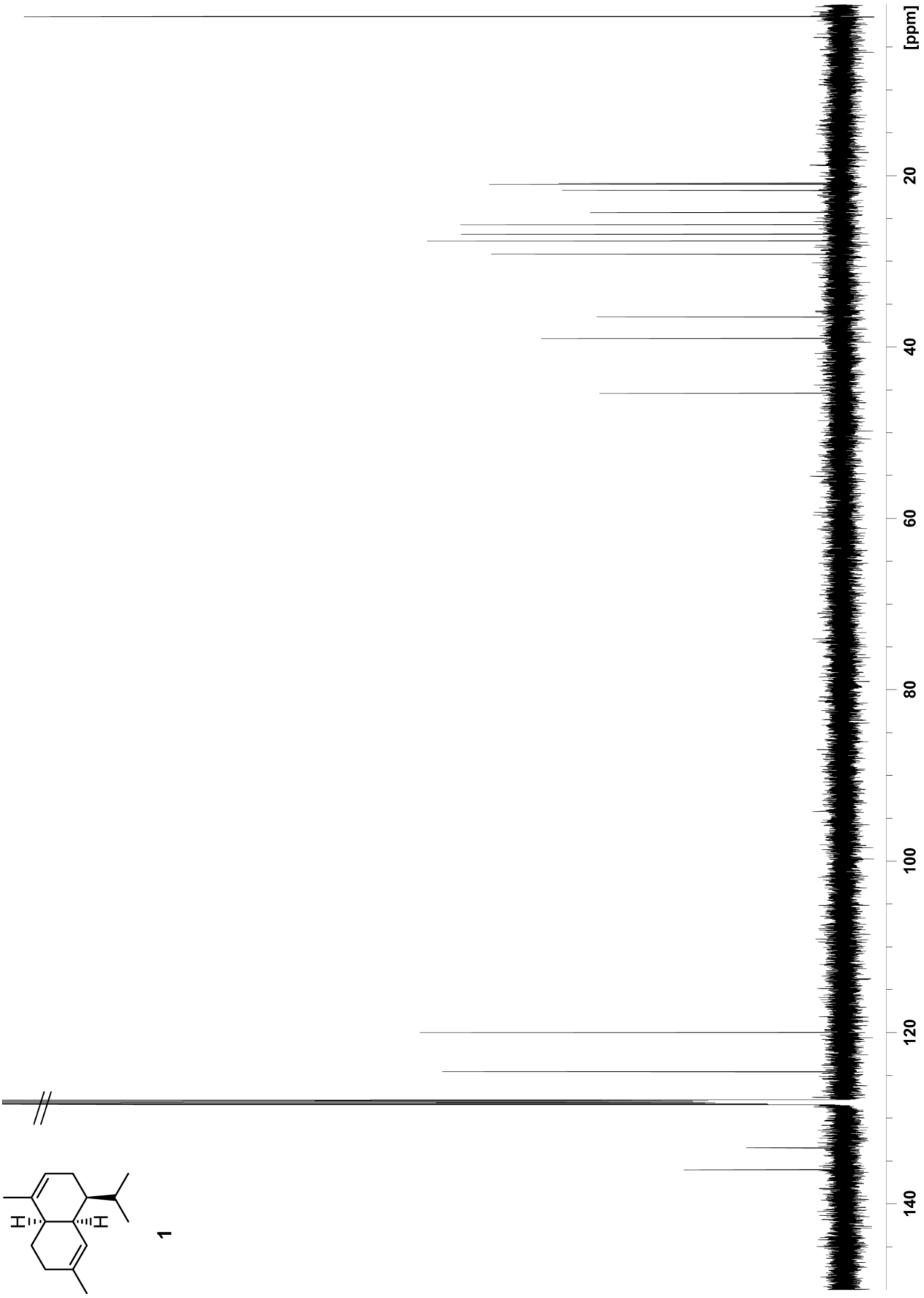

Figure S3: ^{13}C NMR spectrum of **1** at 125 MHz.

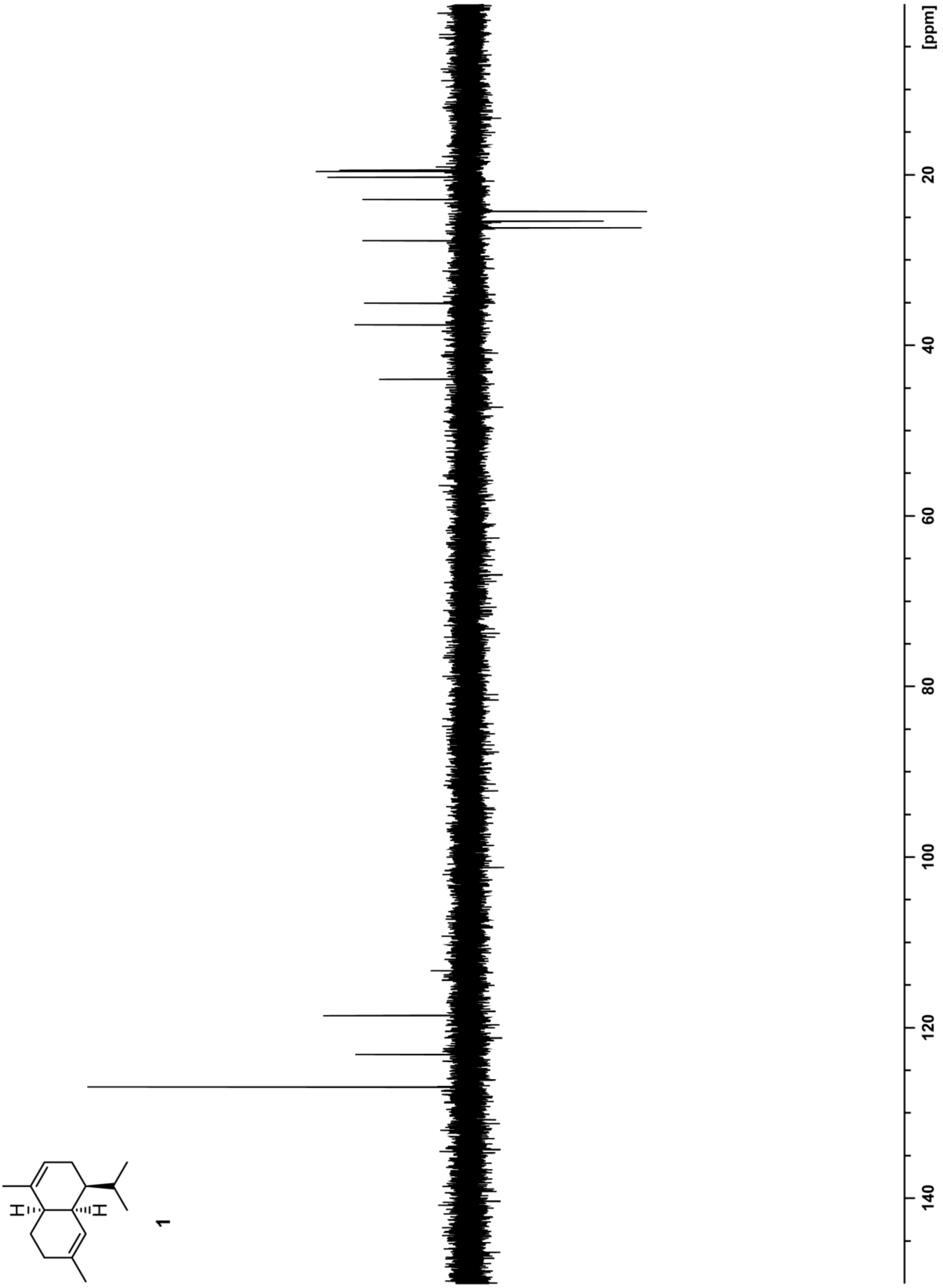

Figure S4: ^{13}C-DEPT135 spectrum of **1** at 125 MHz.

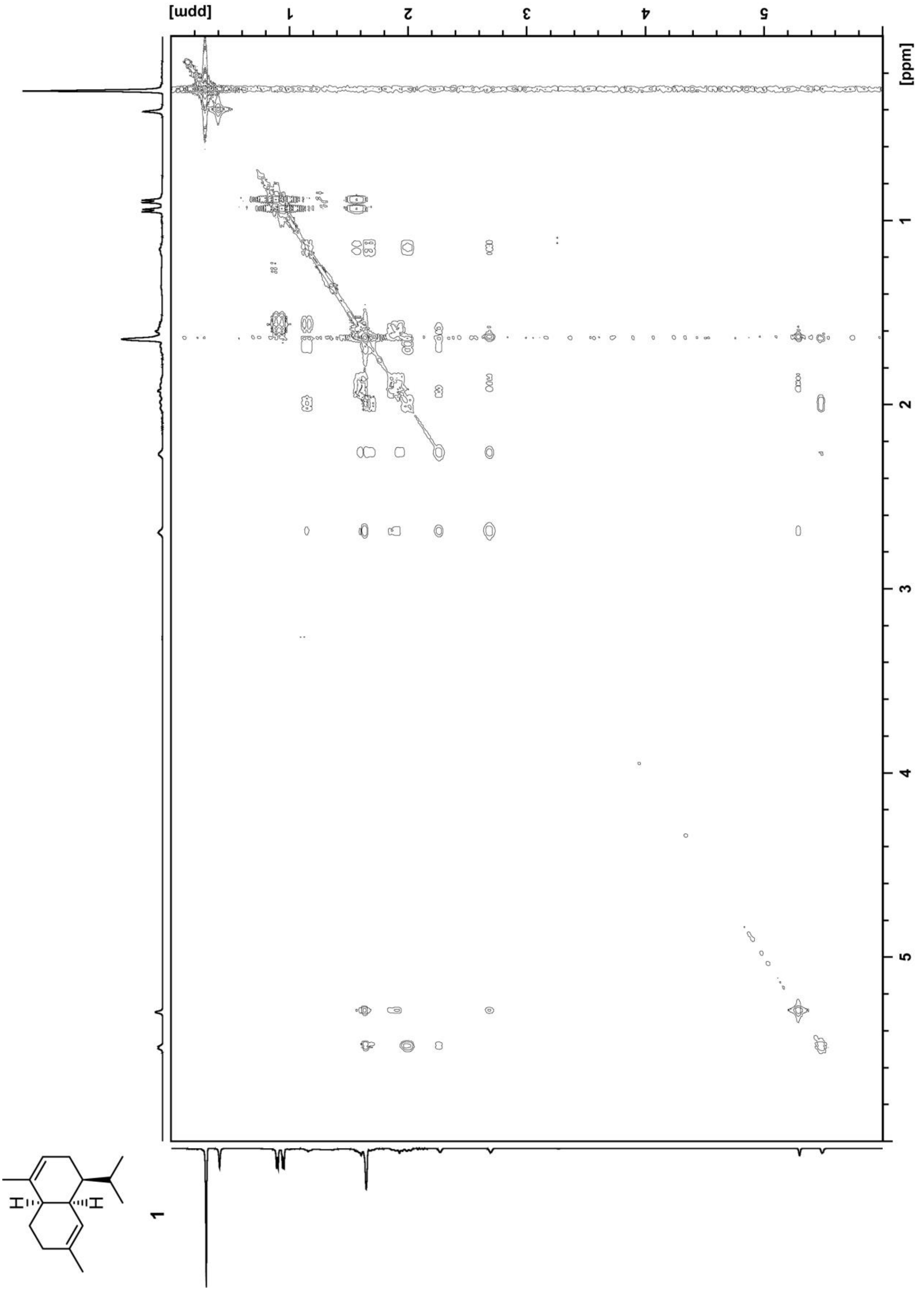

Figure S5: ^{1}H,^{1}H-COSY spectrum of **1**.

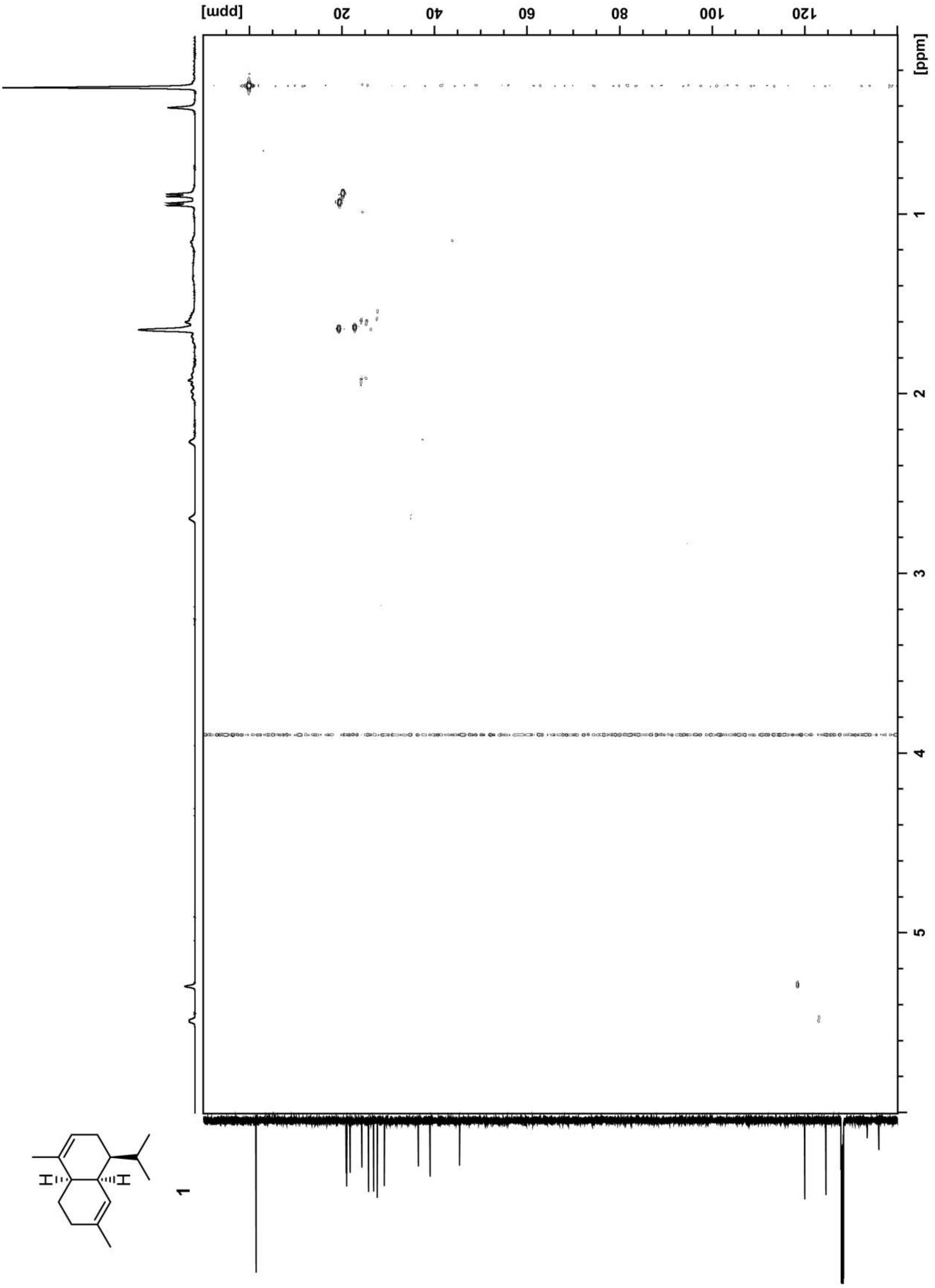

Figure S6: HSQC spectrum of **1**.

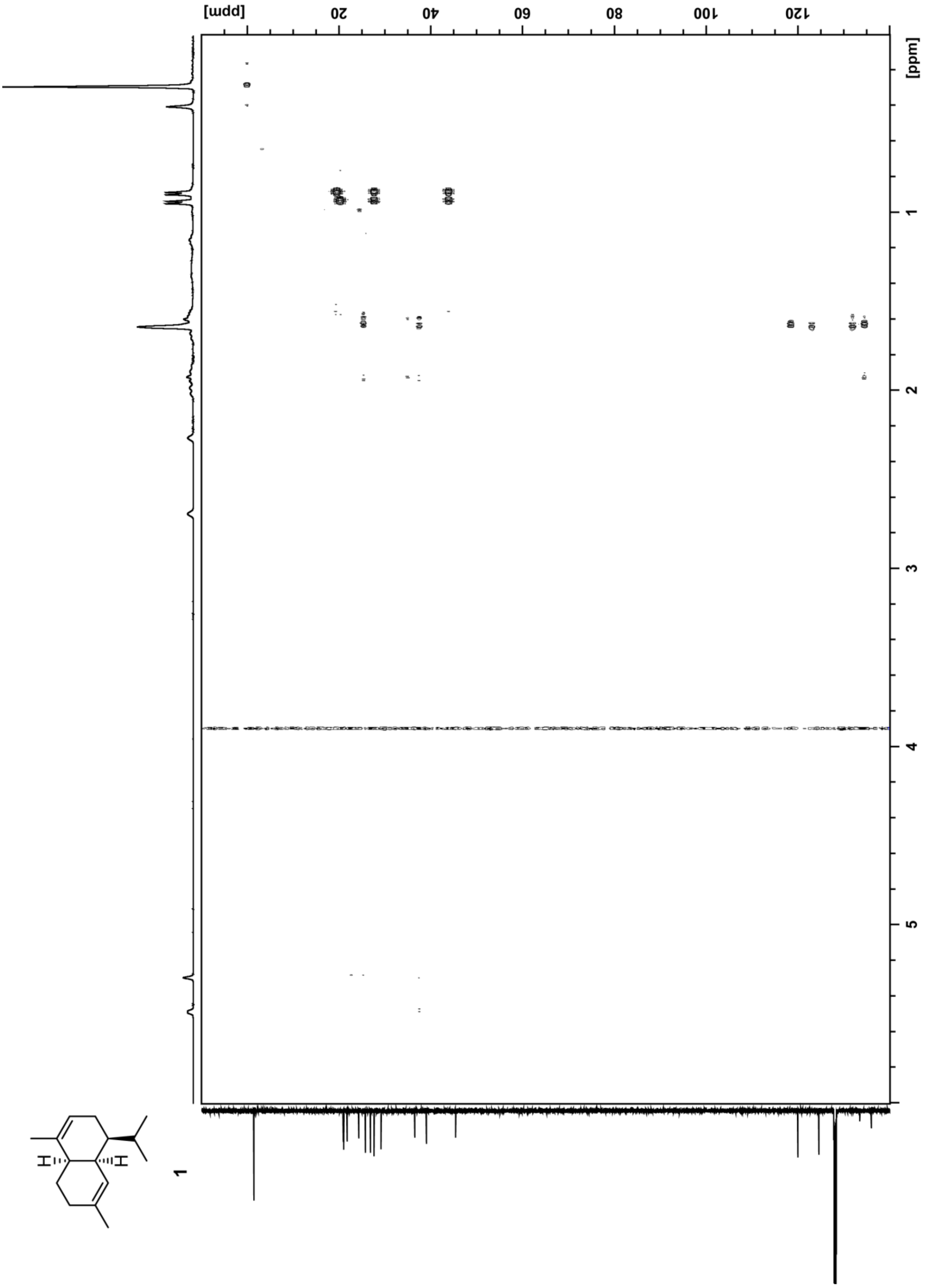

Figure S7: HMBC spectrum of **1**.

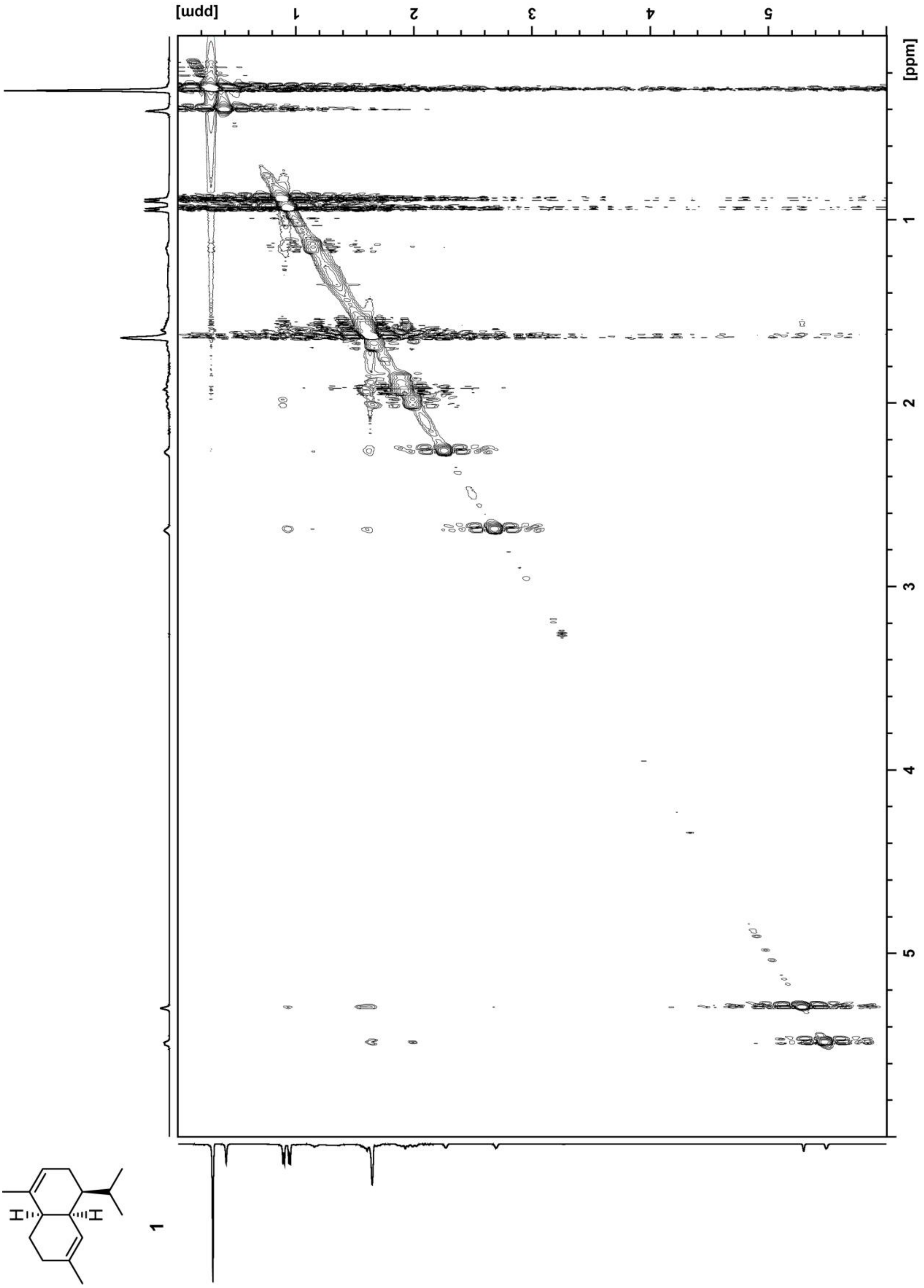

Figure S8: NOESY spectrum of **1**.

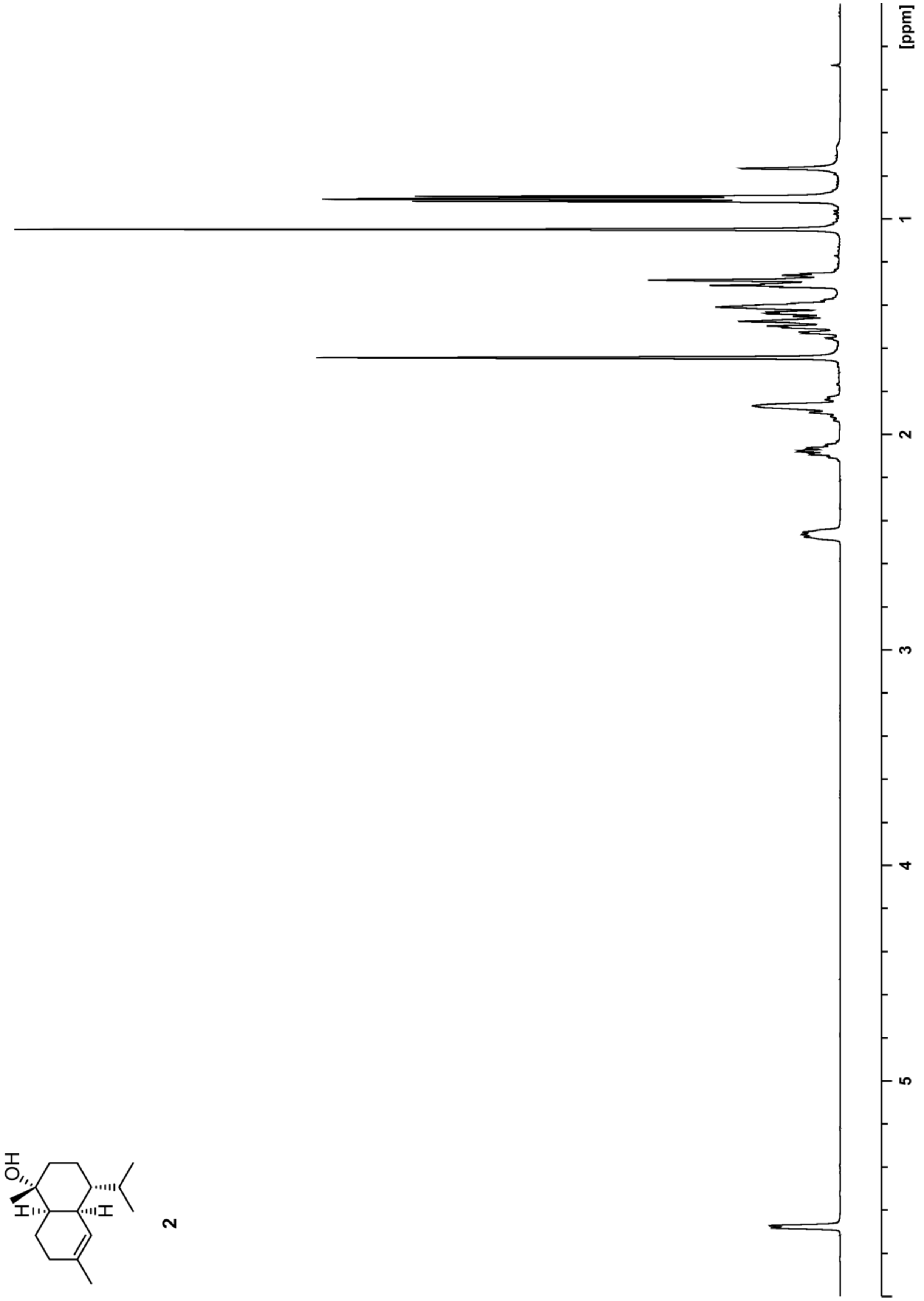

Figure S9: ^{1}H NMR spectrum of **2** at 500 MHz.

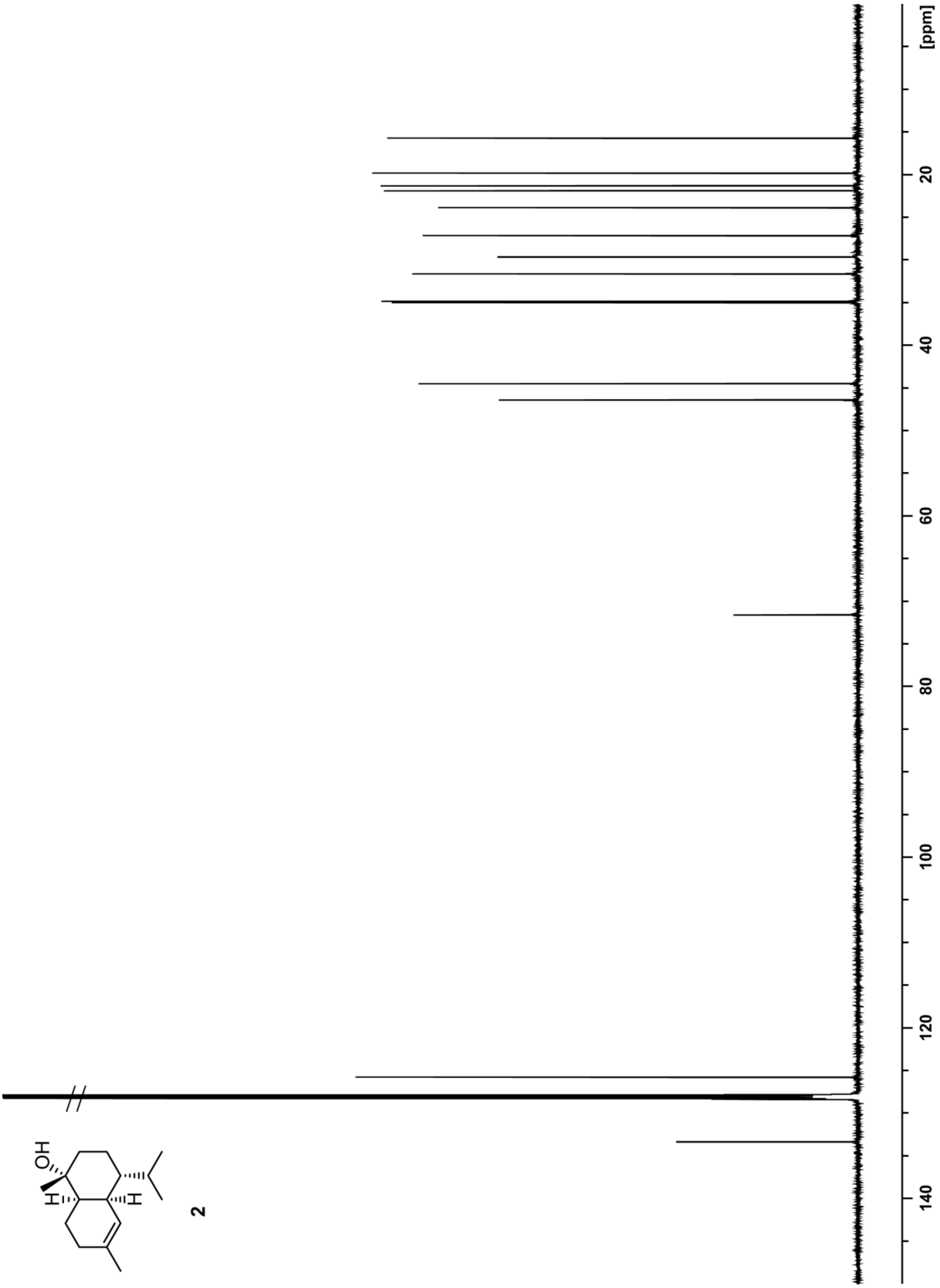

Figure S10: ^{13}C NMR spectrum of **2** at 125 MHz.

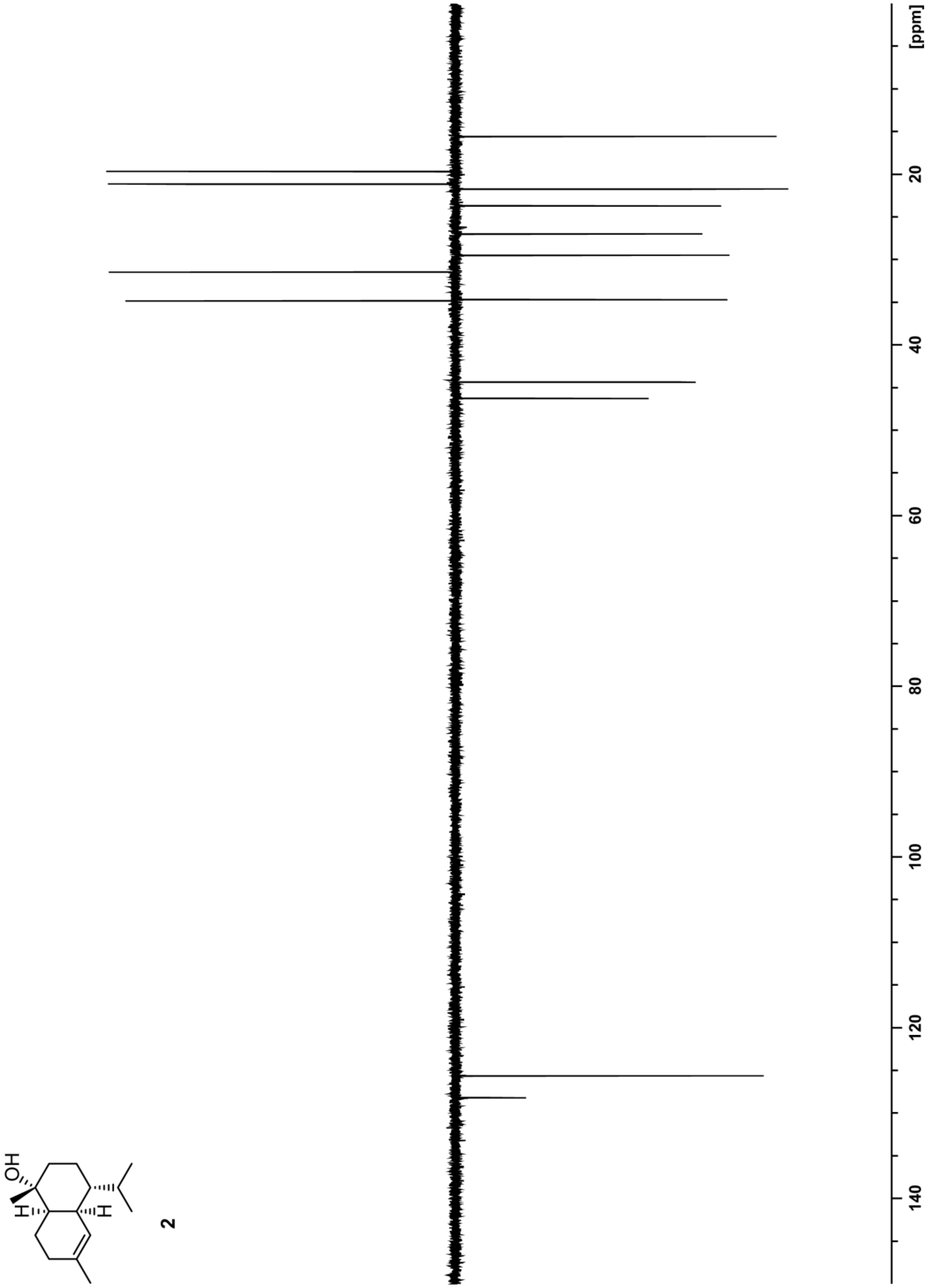

Figure S11: ^{13}C-DEPT135 spectrum of **2** at 125 MHz.

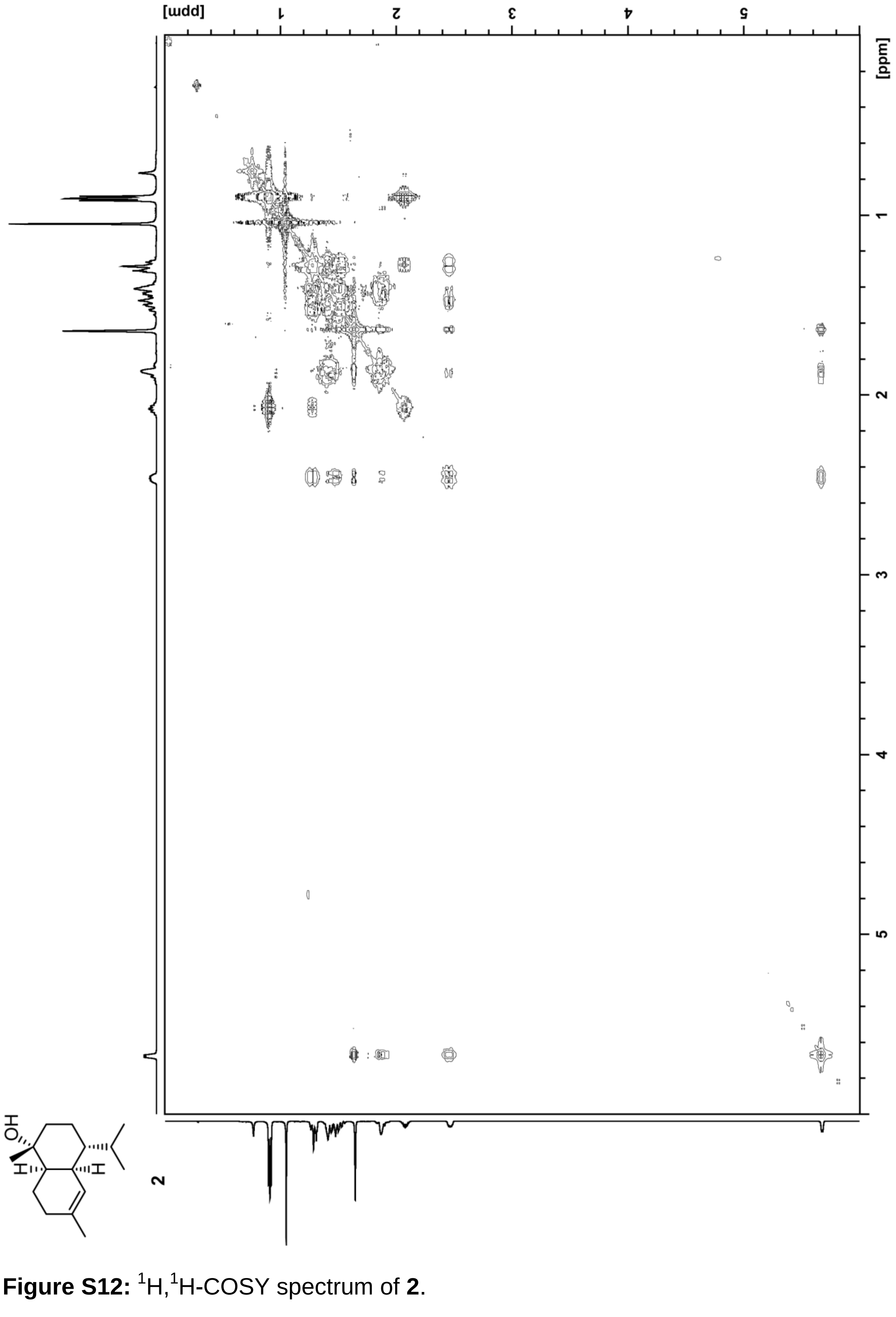

2

Figure S12: $^{1}H,^{1}H$-COSY spectrum of **2**.

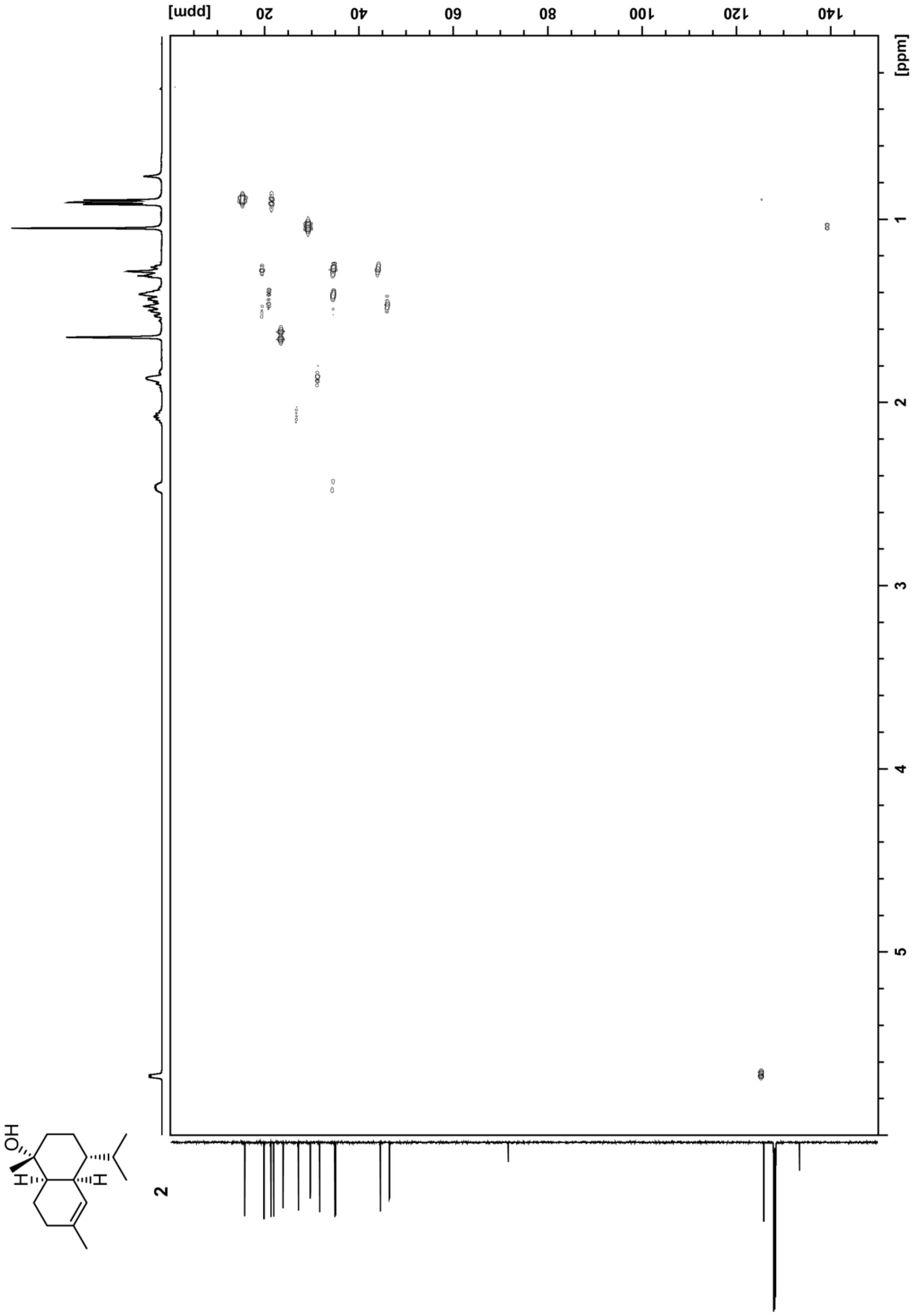

Figure S13: HSQC spectrum of **2**.

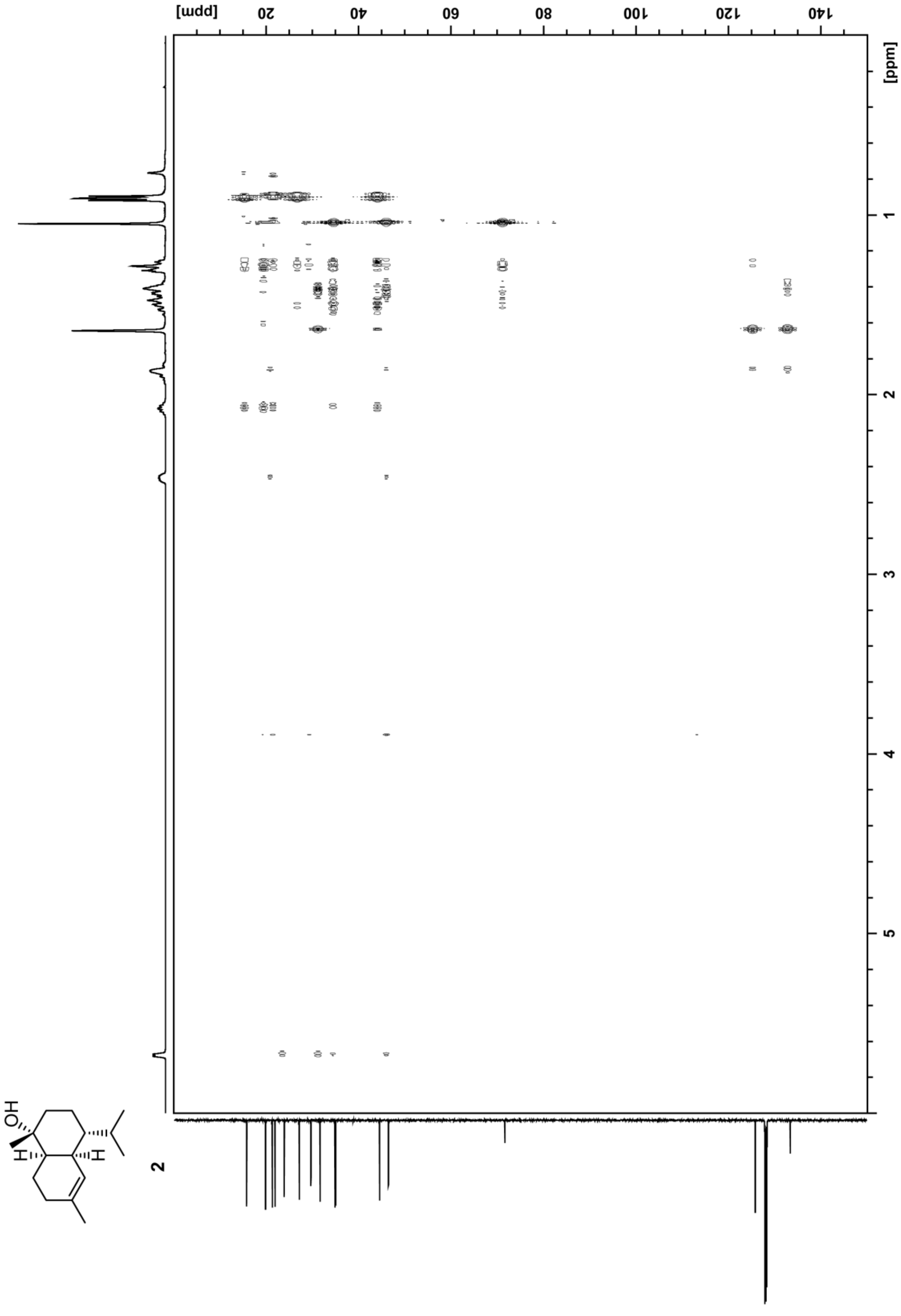

Figure S14: HMBC spectrum of **2**.

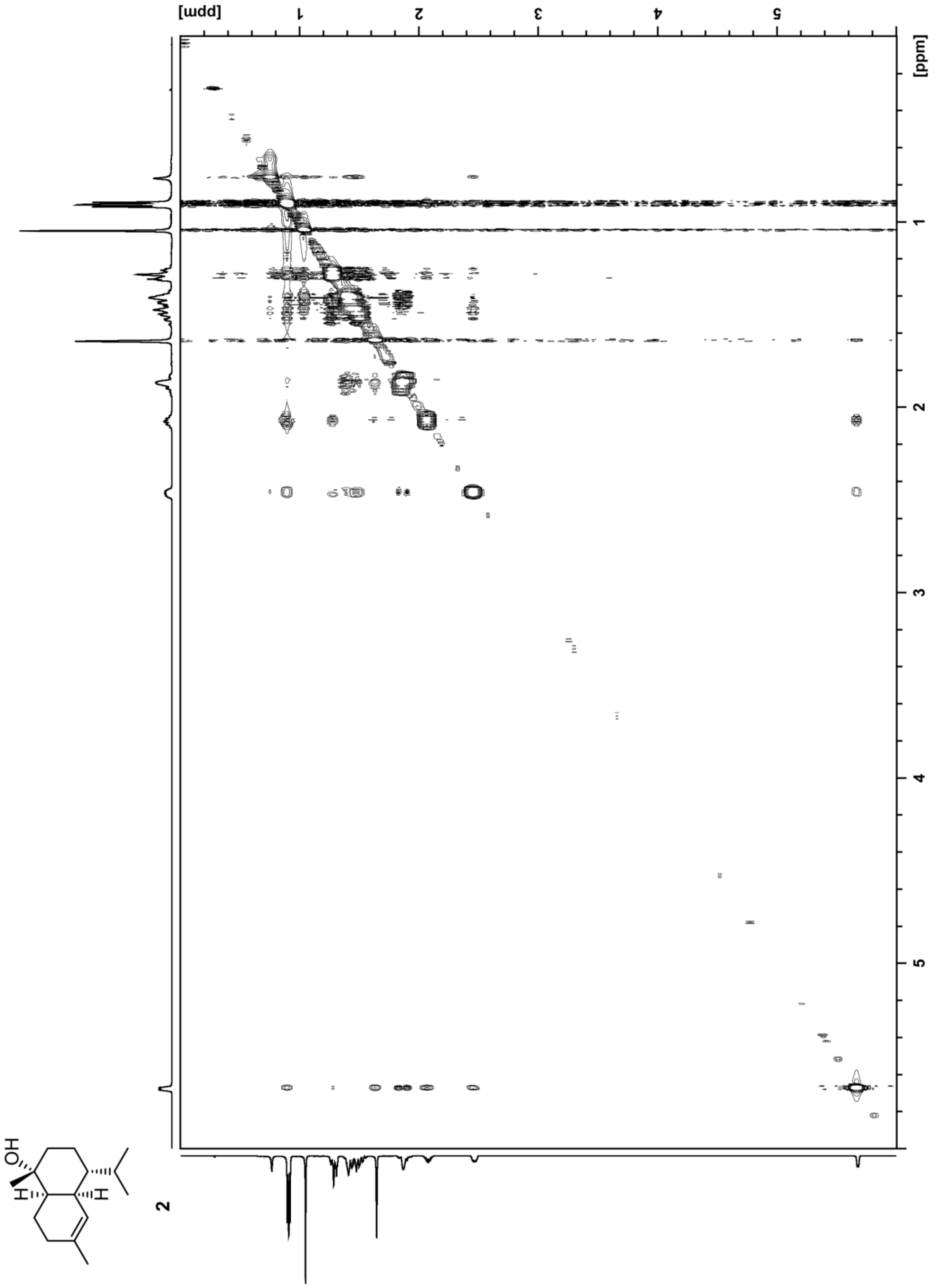

Figure S15: NOESY spectrum of **2**.

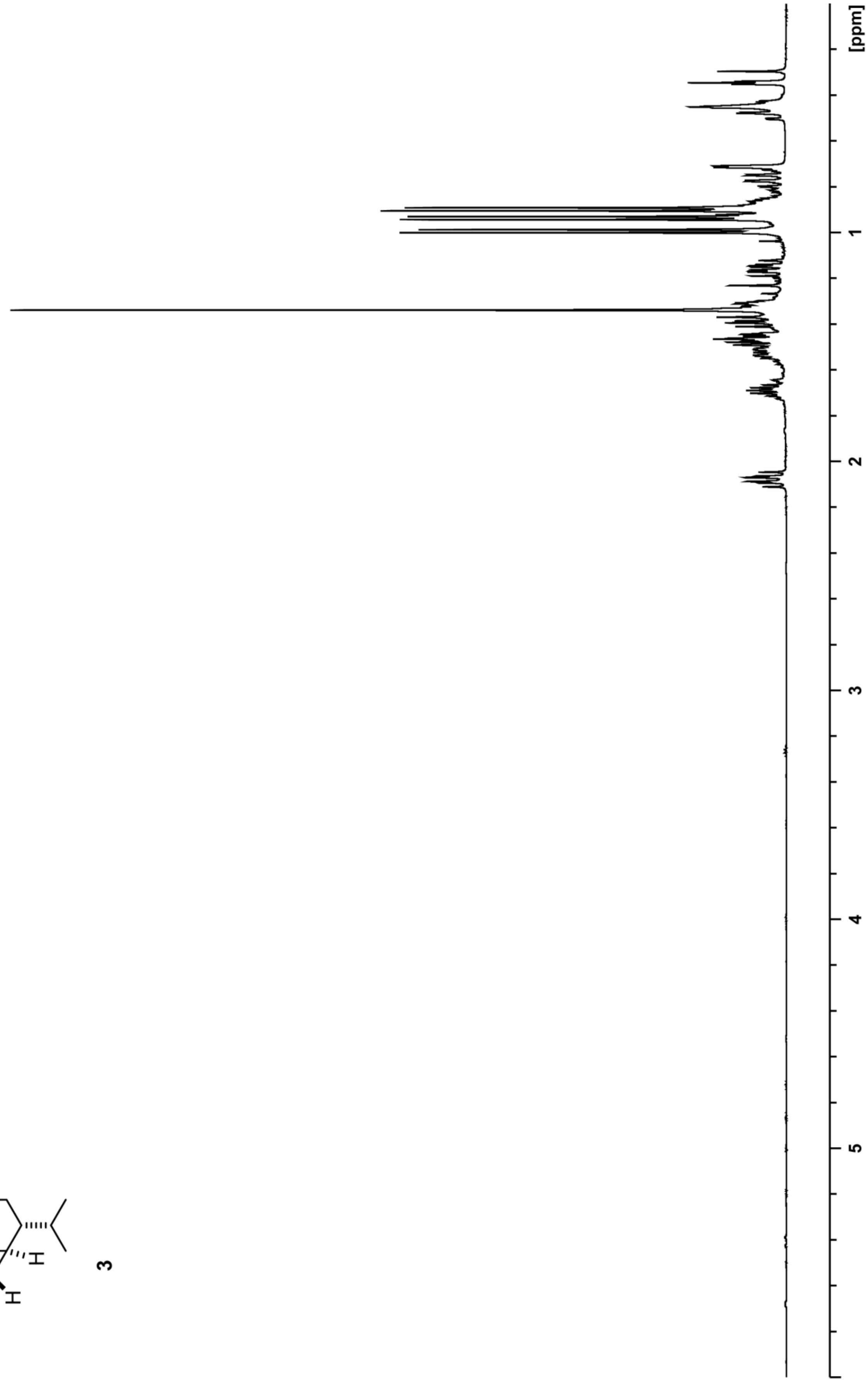

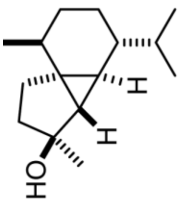

3

Figure S16: ^{1}H NMR spectrum of **3** at 500 MHz.

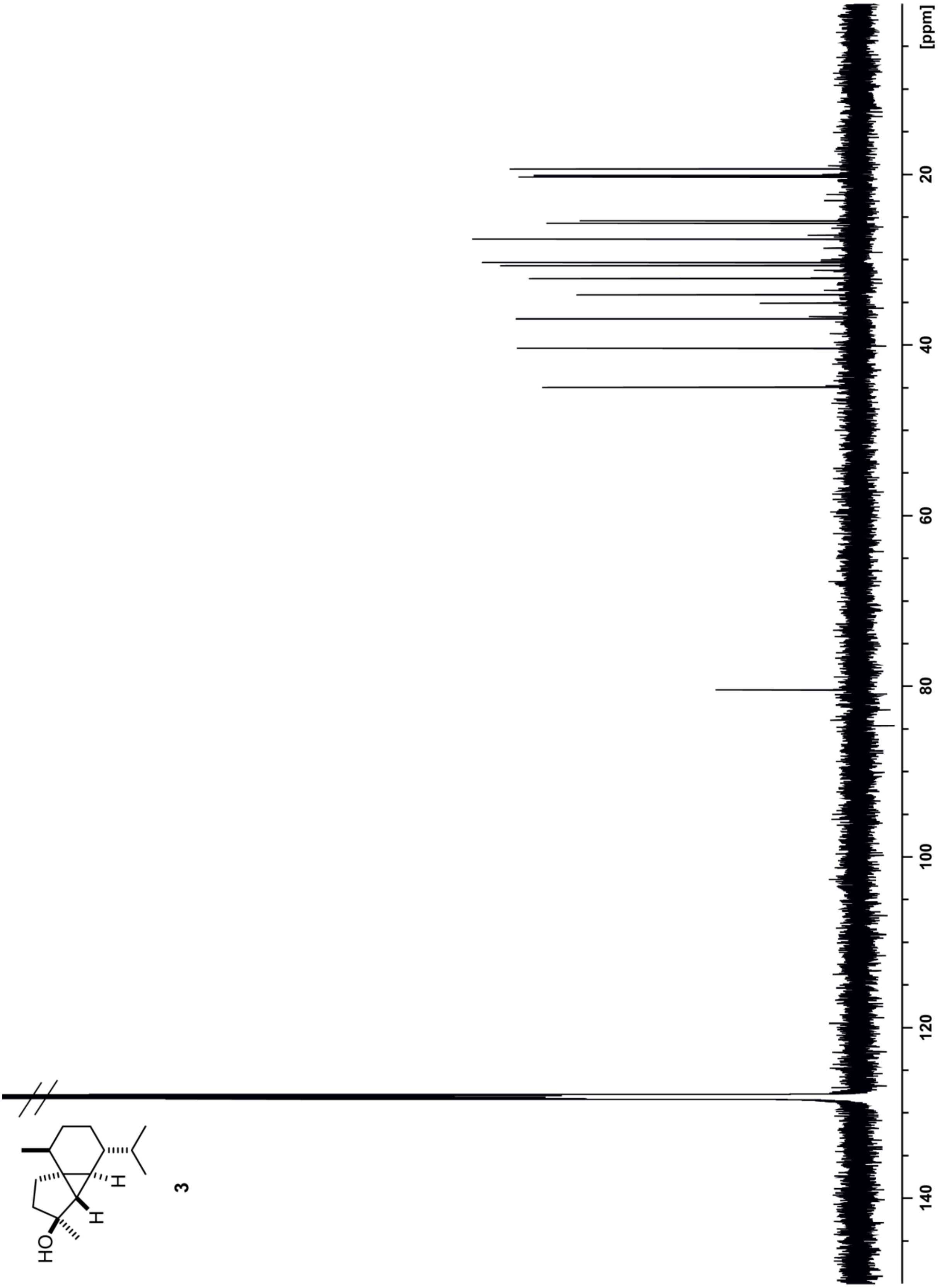

Figure S17: ^{13}C NMR spectrum of **3** at 125 MHz.

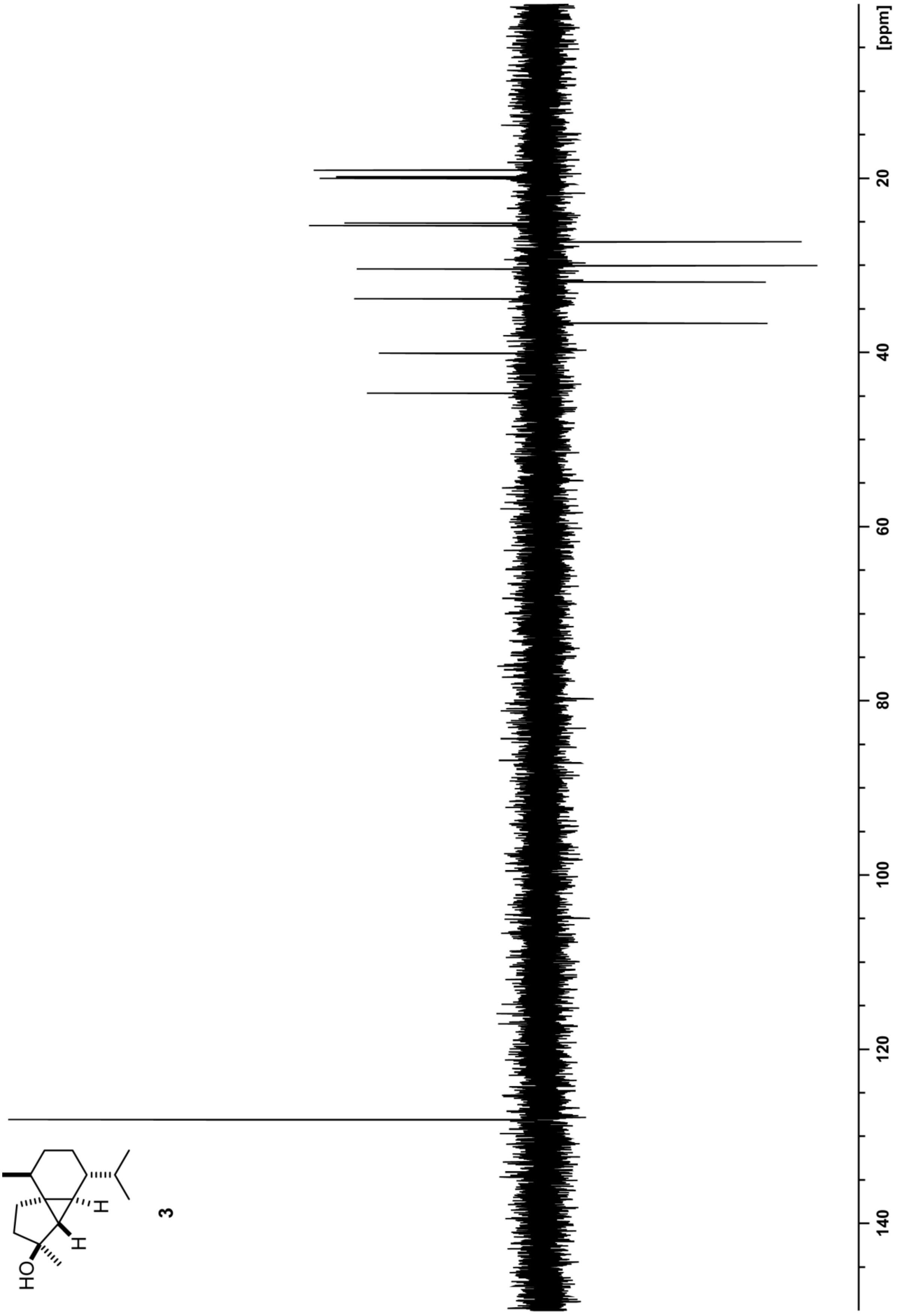

Figure S18: ^{13}C-DEPT135 NMR spectrum of **3** at 125 MHz.

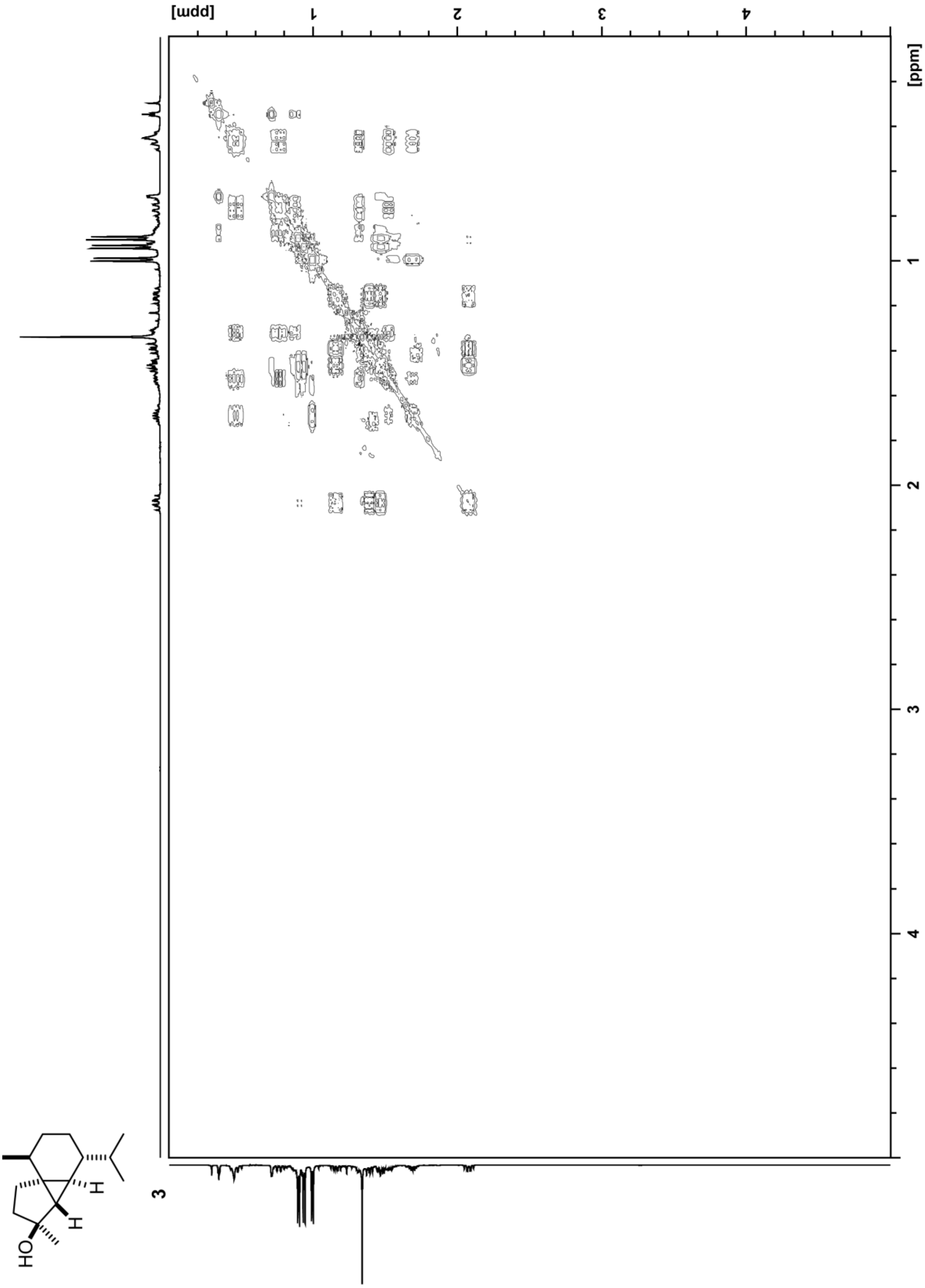

Figure S19: $^{1}H,^{1}H$-COSY NMR spectrum of **3**.

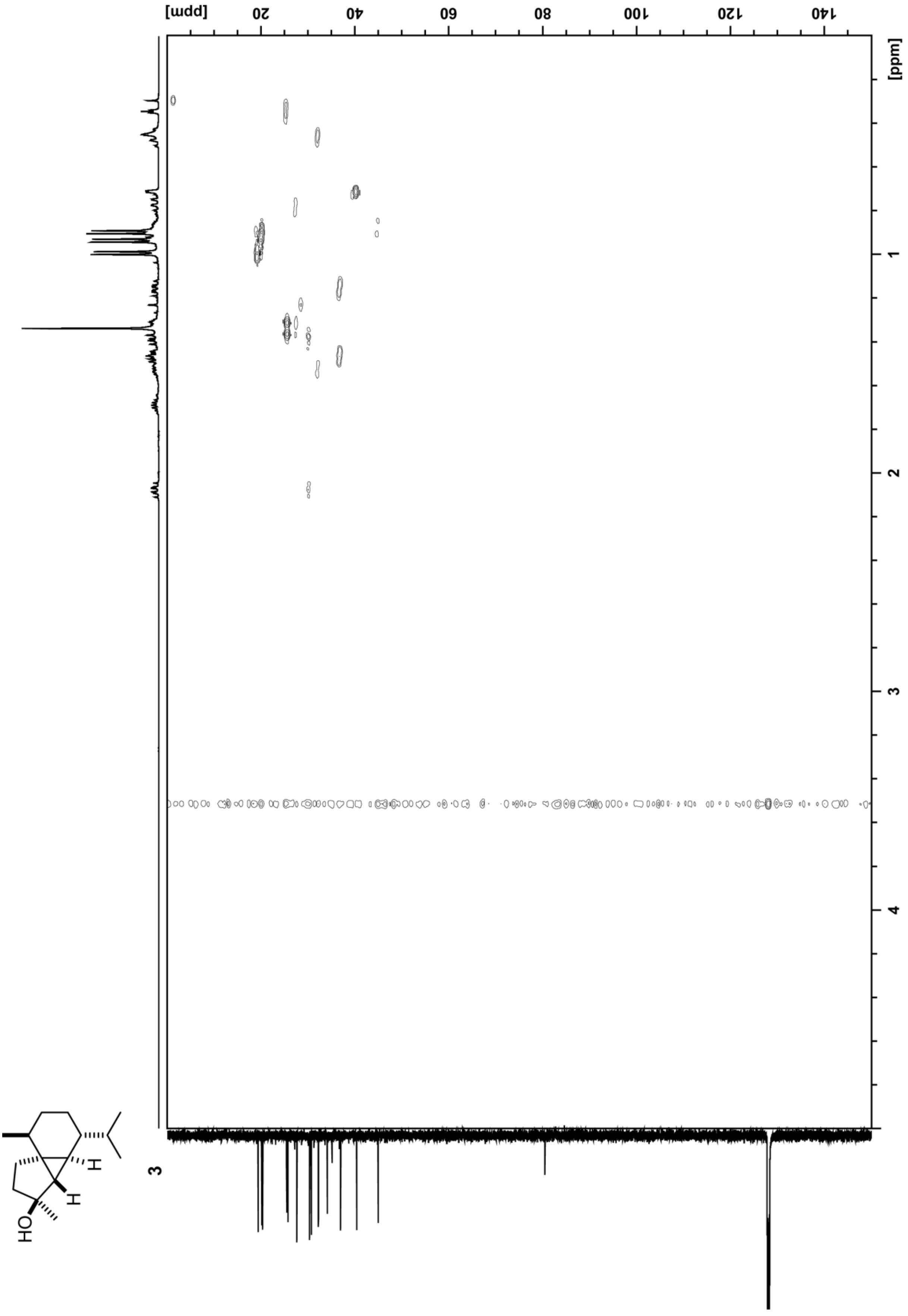

Figure S20: HSQC spectrum of **3**.

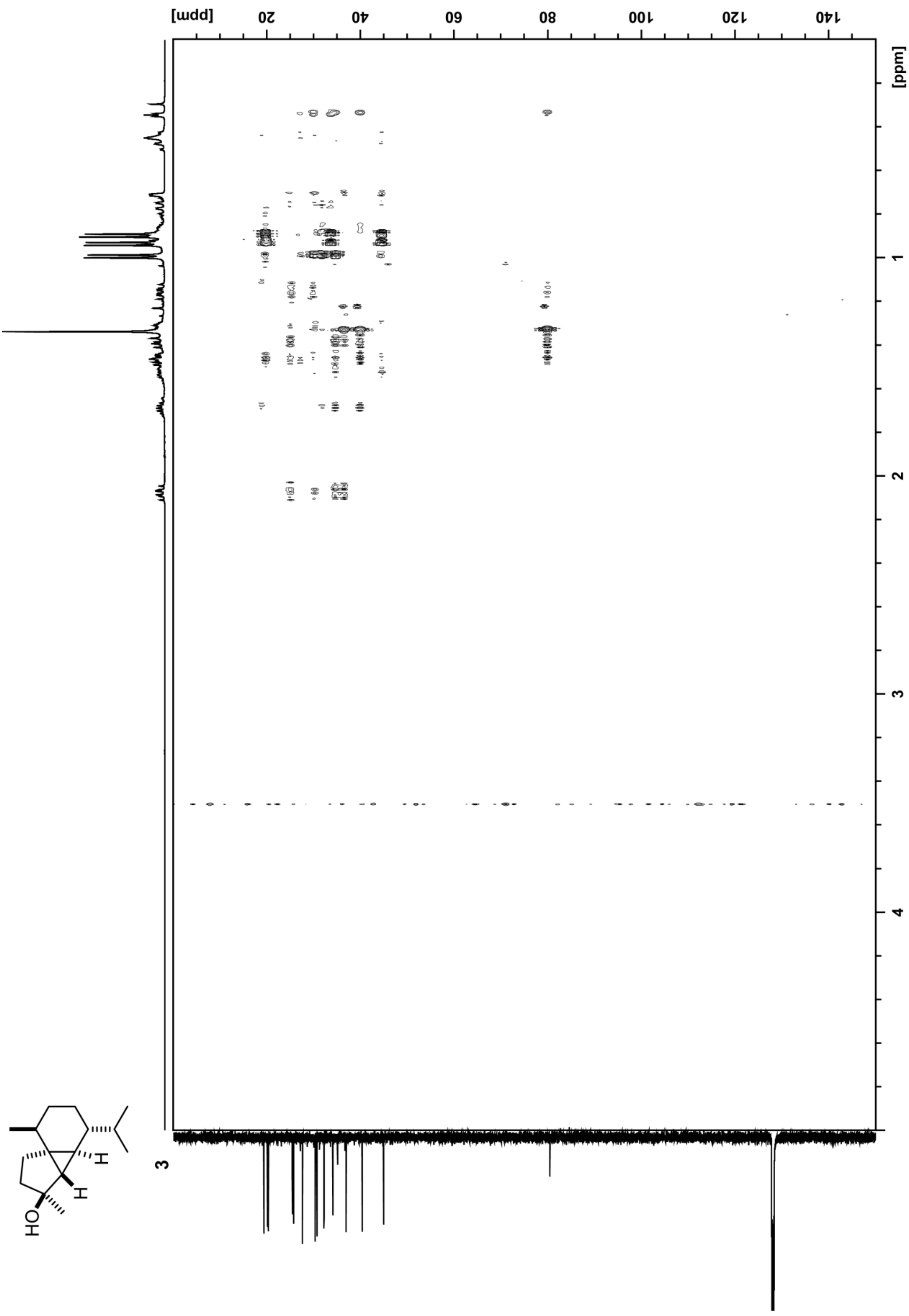

Figure S21: HMBC spectrum of **3**.

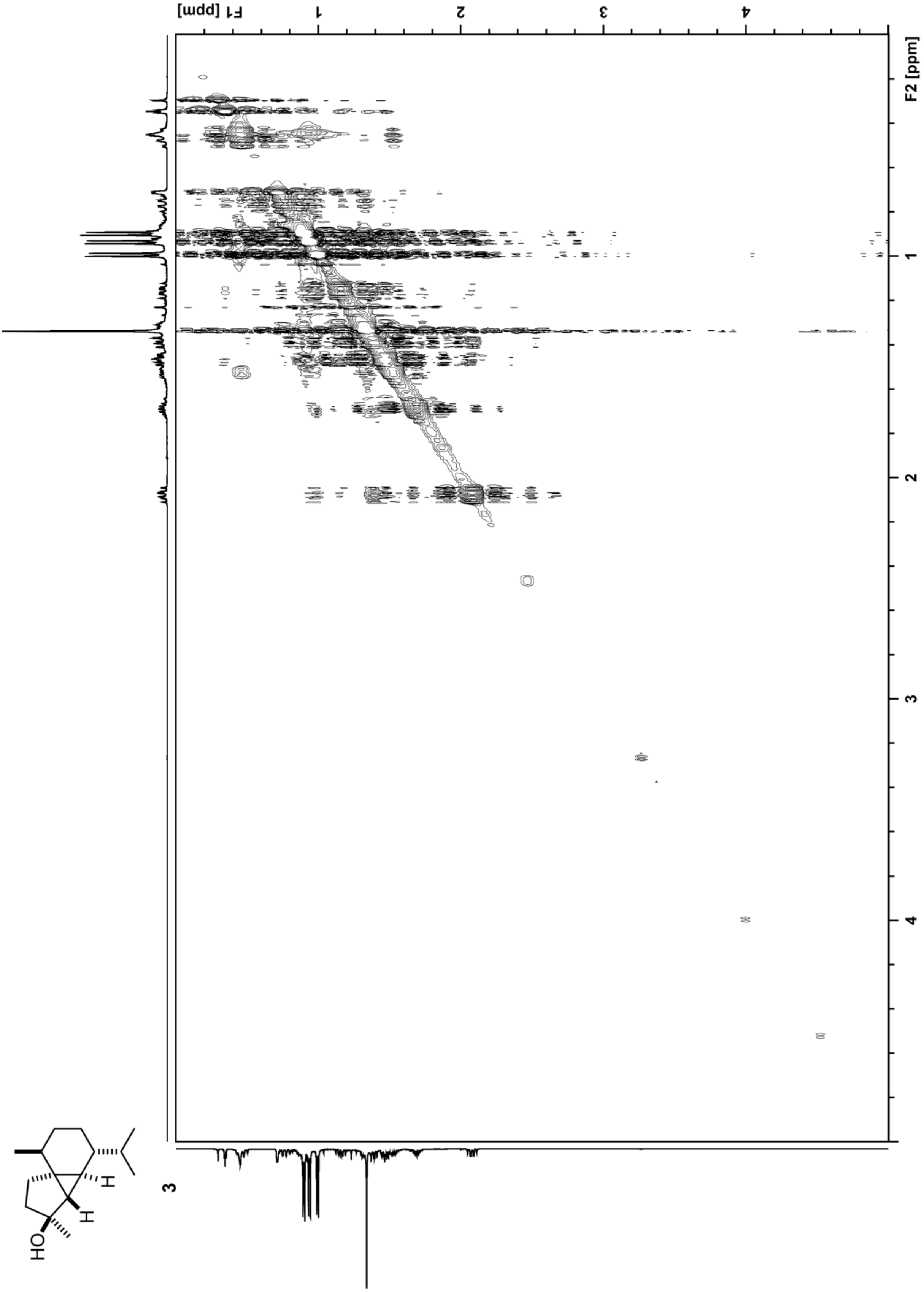

3

Figure S22: NOESY spectrum of **3**.

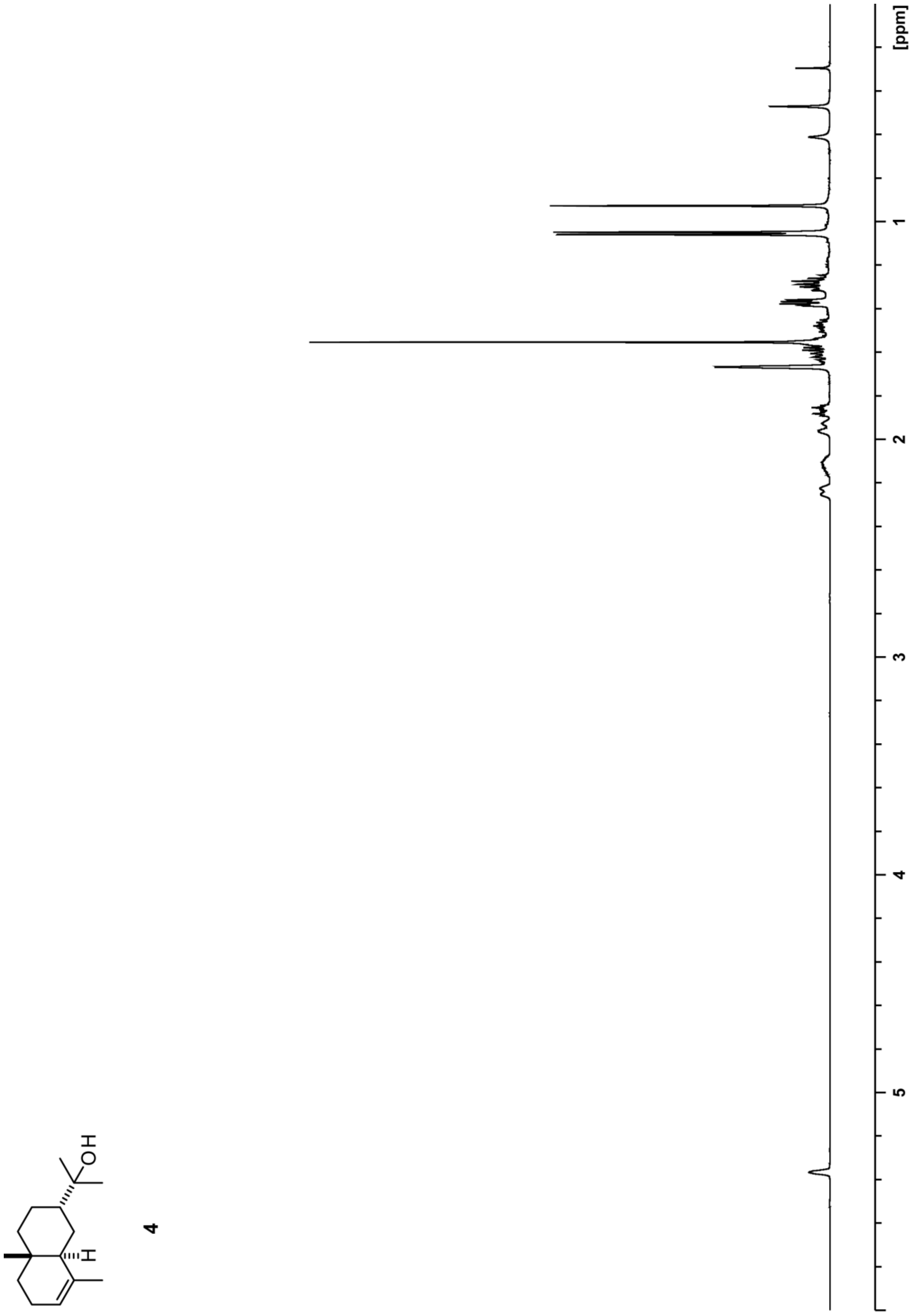

Figure S23: ^{1}H NMR spectrum of **4** at 500 MHz.

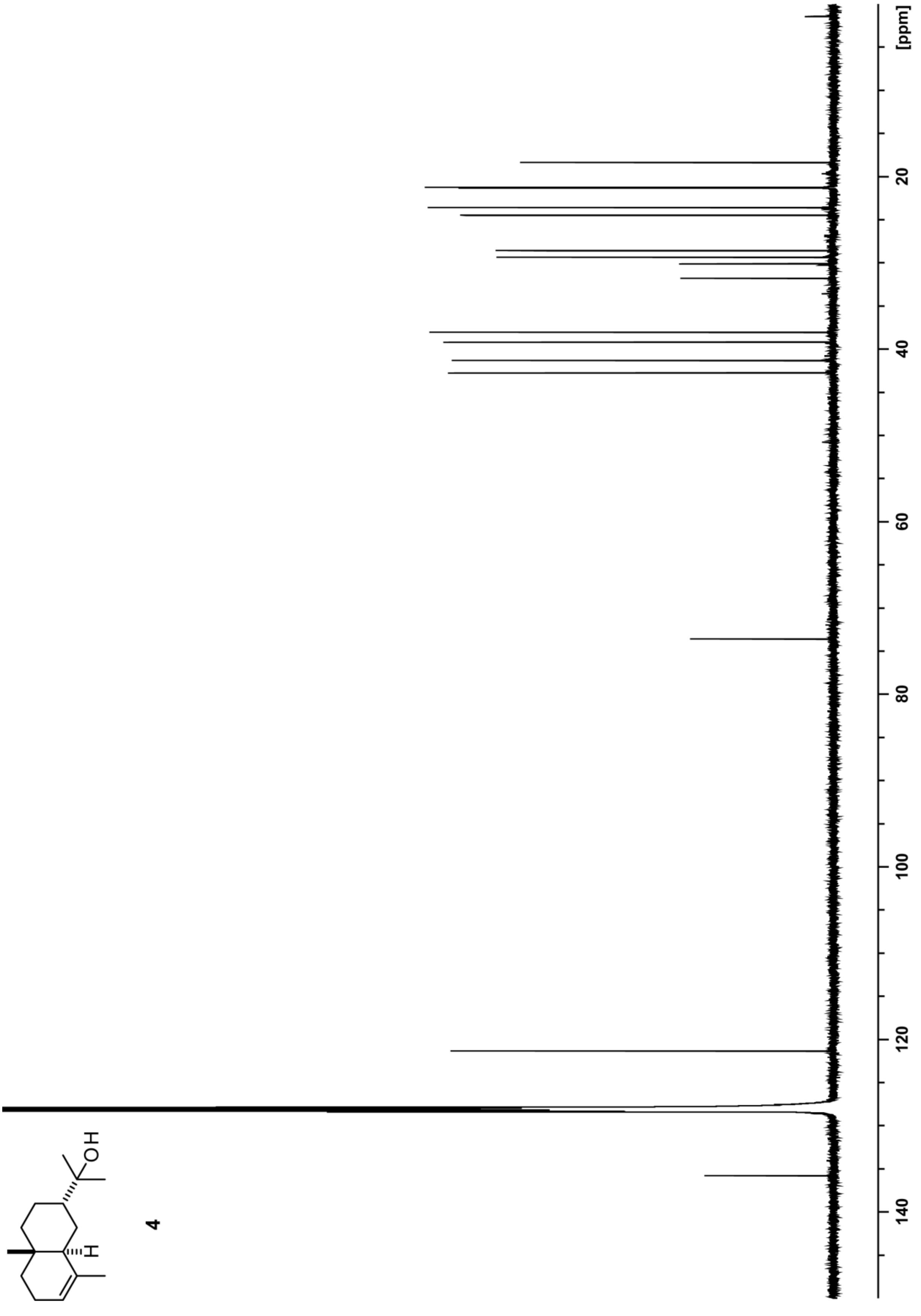

Figure S24: ^{13}C-NMR spectrum of **4** at 125 MHz.

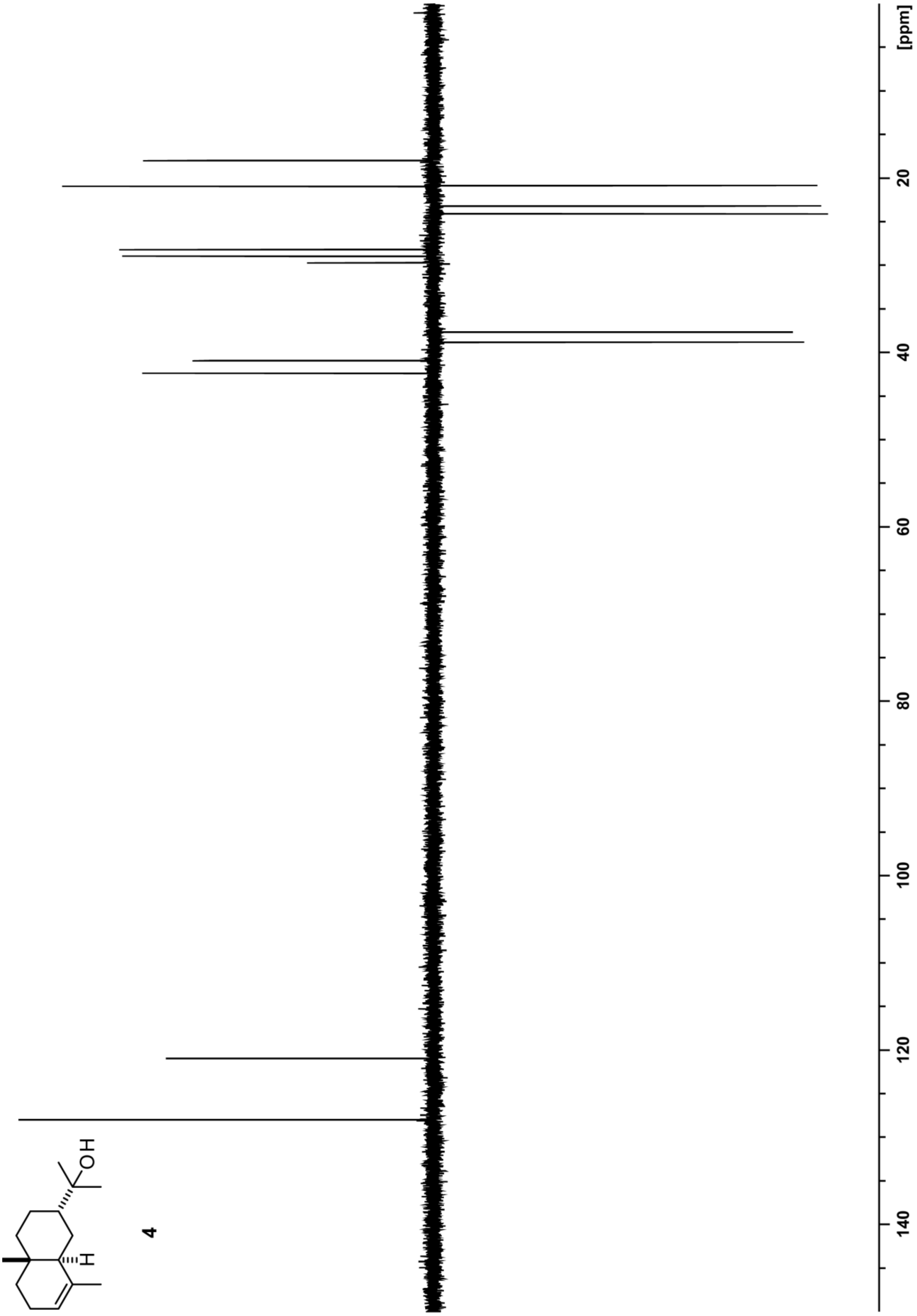

Figure S25: ^{13}C-DEPT135 NMR spectrum of **4** at 125 MHz.

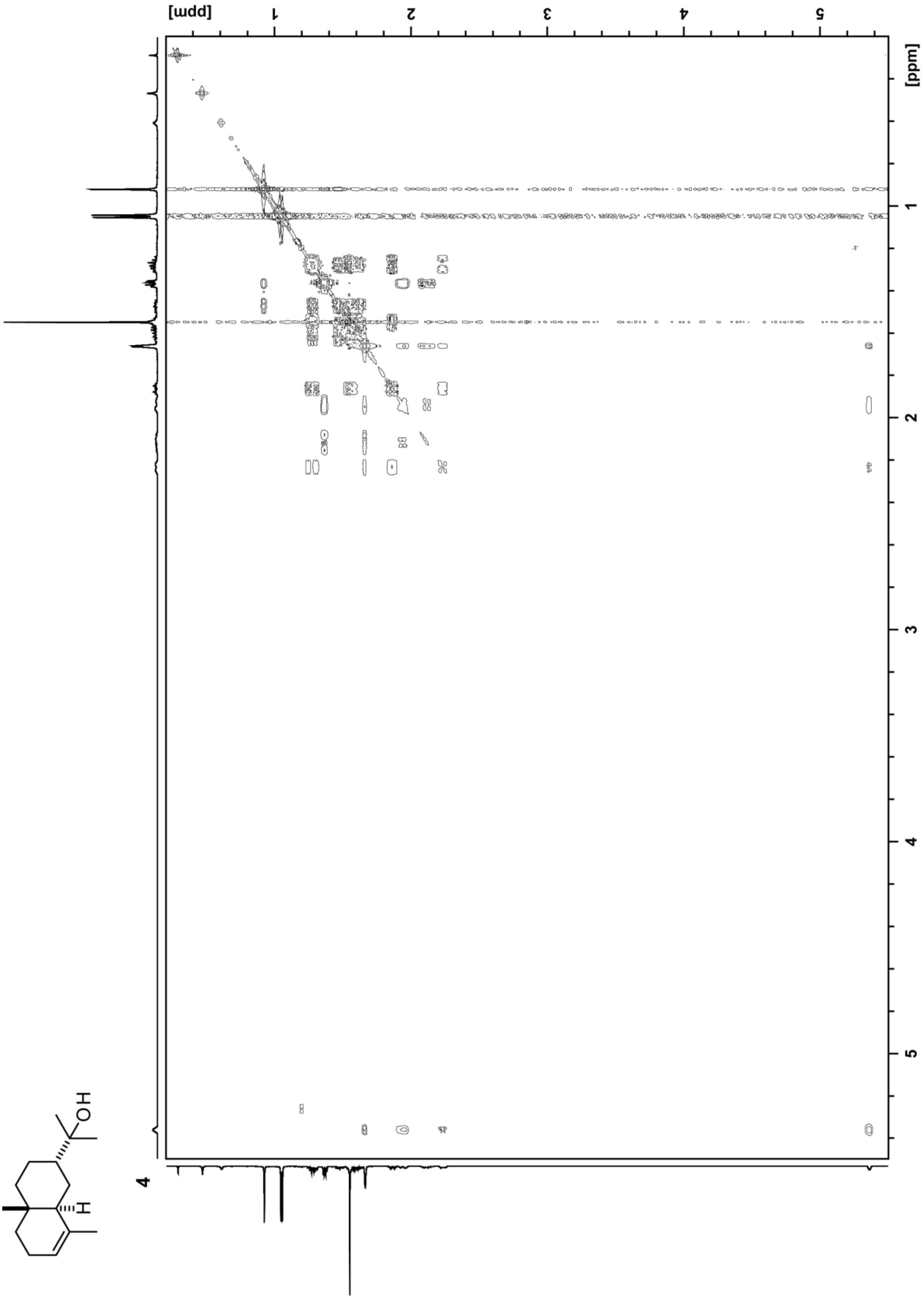

Figure S26: ^{1}H,^{1}H-COSY spectrum of **4**.

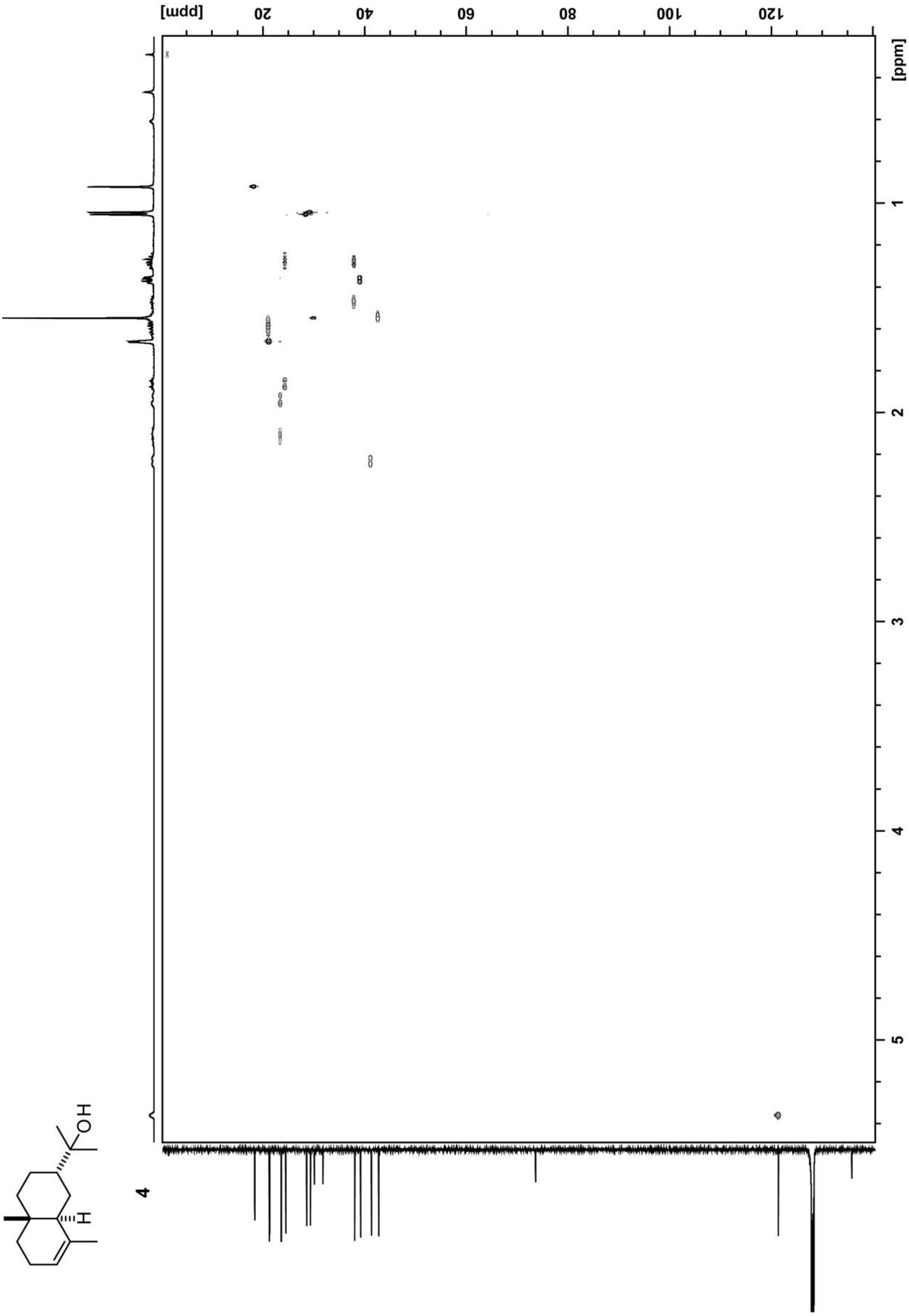

Figure S27: HSQC spectrum of **4**.

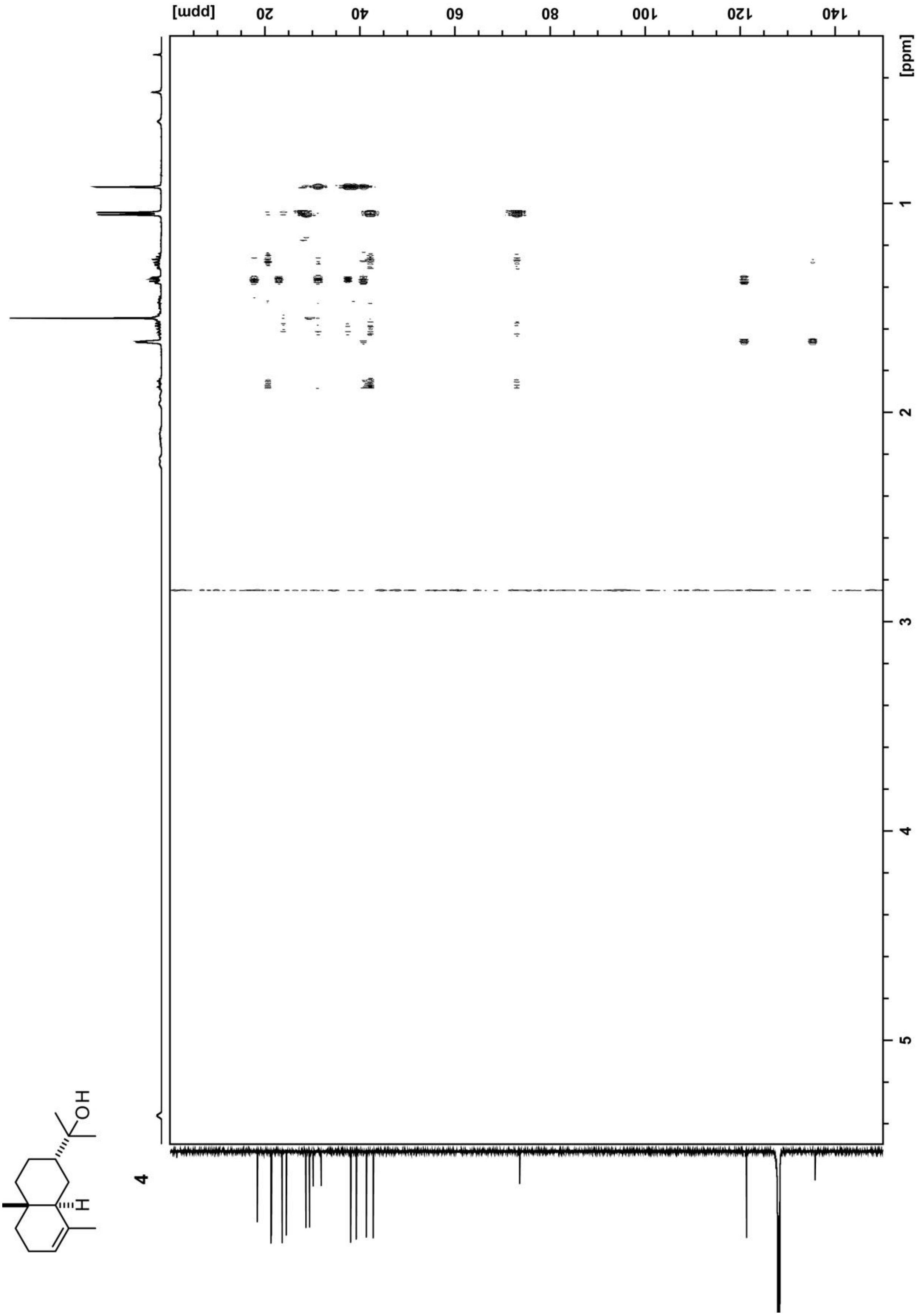

Figure S28: HMBC spectrum of **4**.

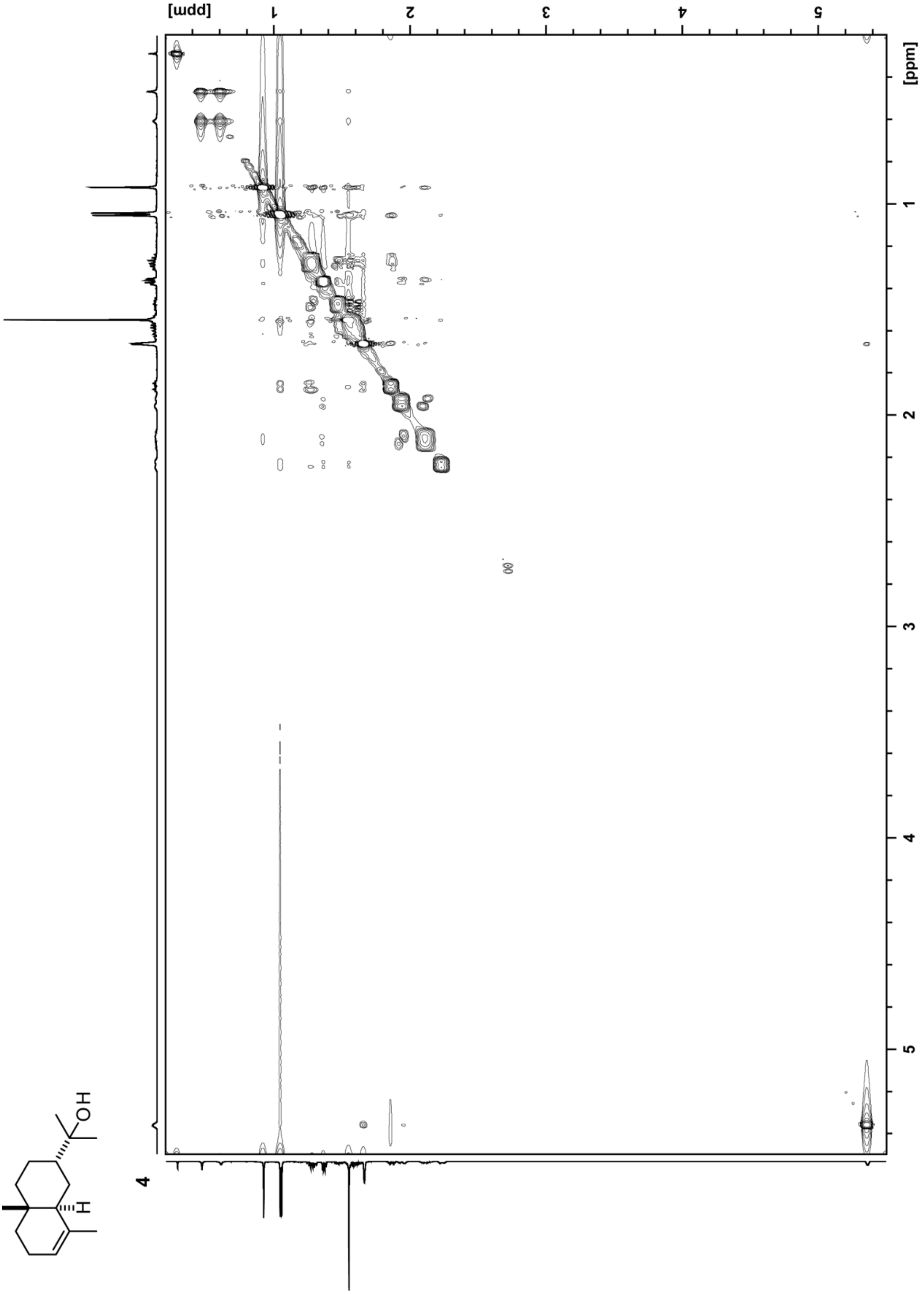

Figure S29: NOESY spectrum of **4**.

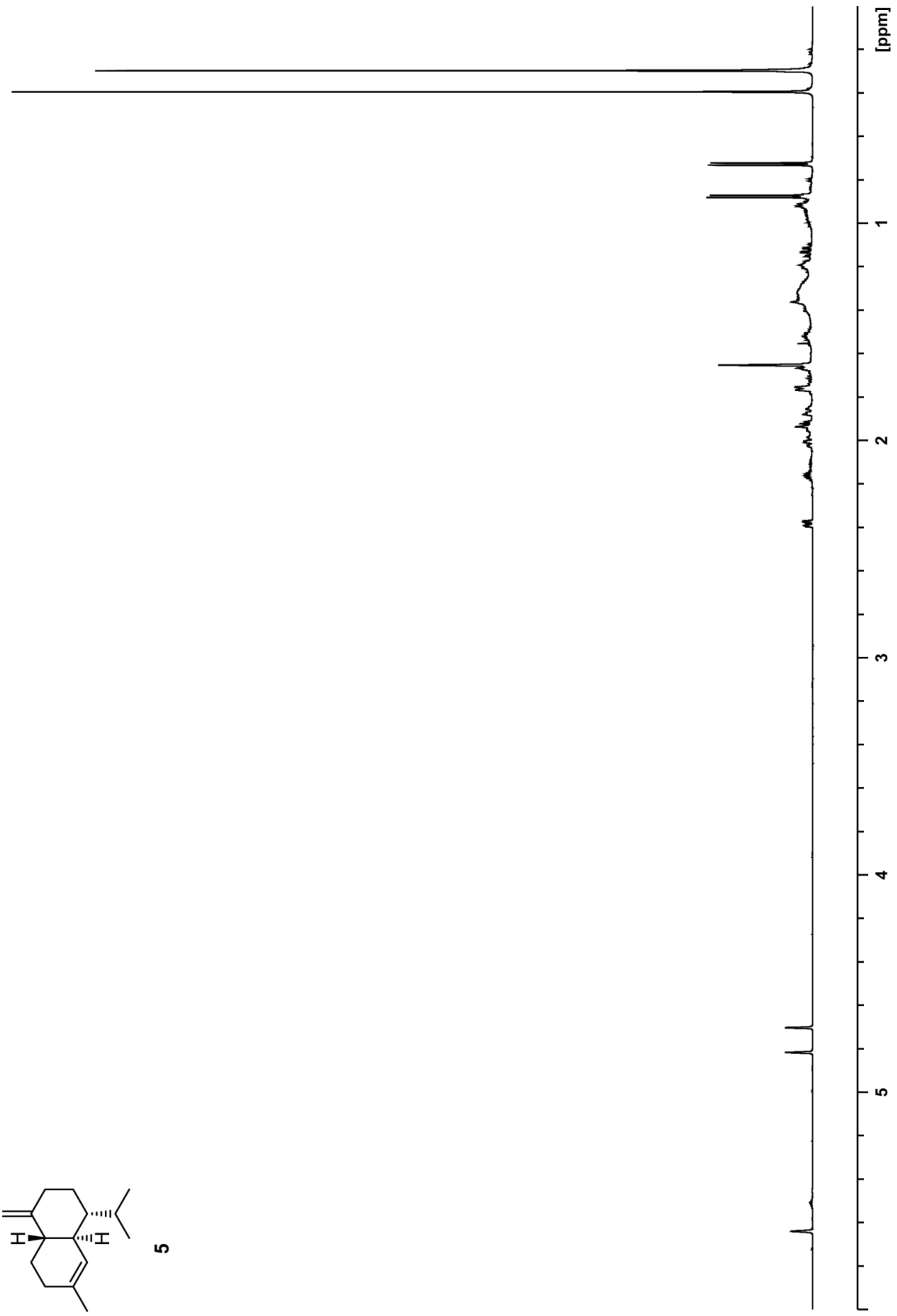

Figure S30: ^{1}H NMR spectrum of **5** at 500 MHz.

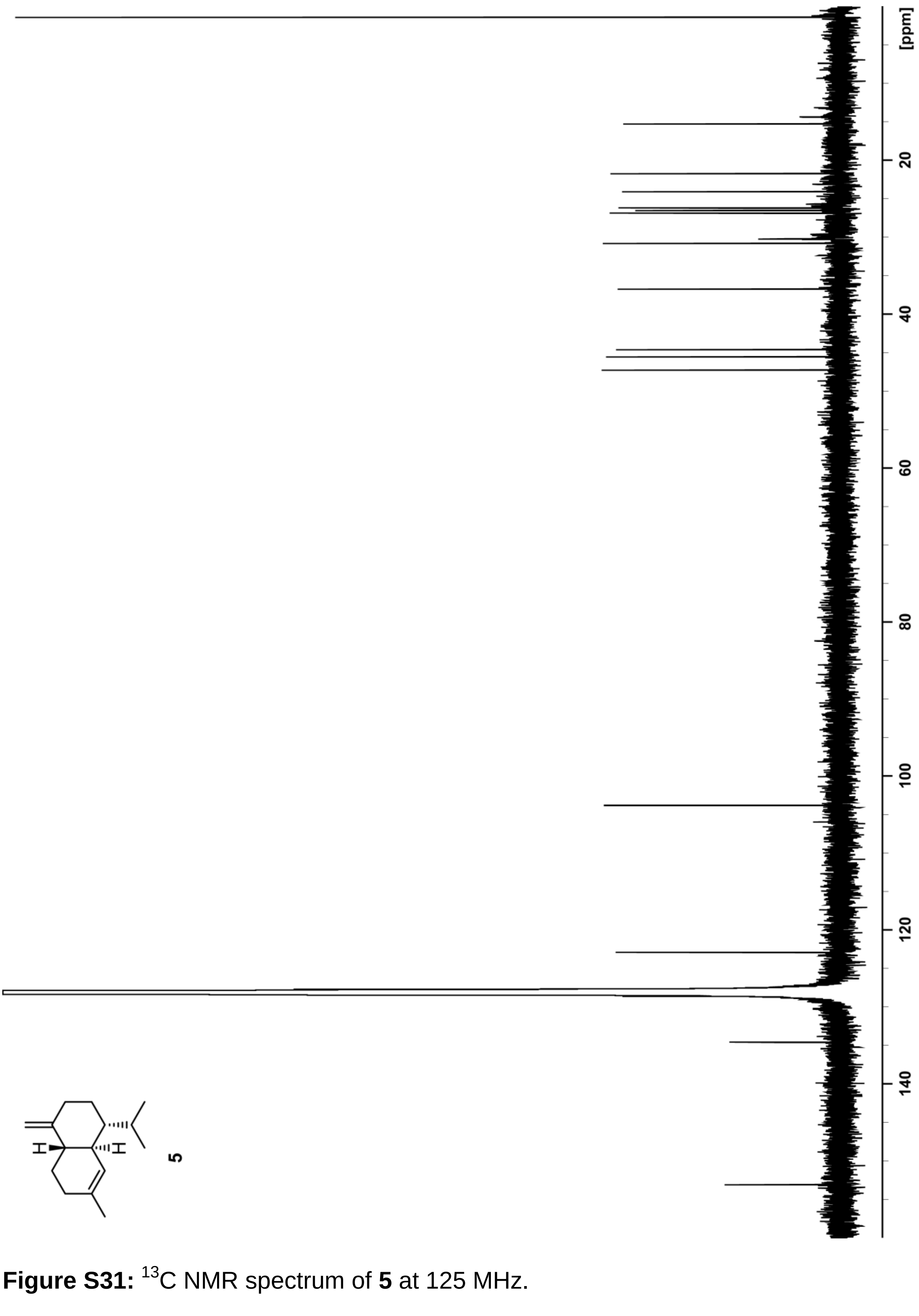

Figure S31: ^{13}C NMR spectrum of **5** at 125 MHz.

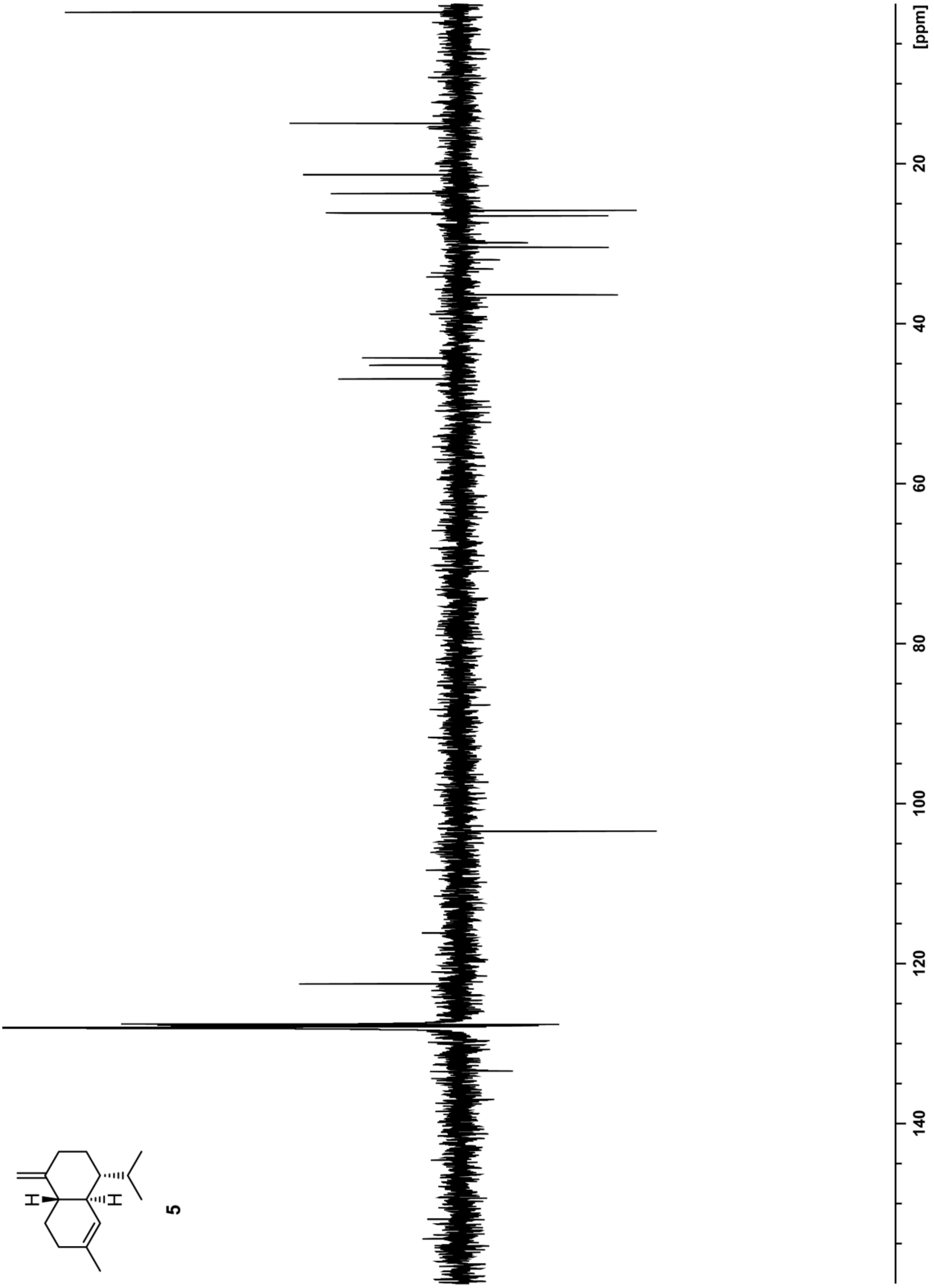

Figure S32: ^{13}C-DEPT135 NMR spectrum of **5** at 125 MHz.

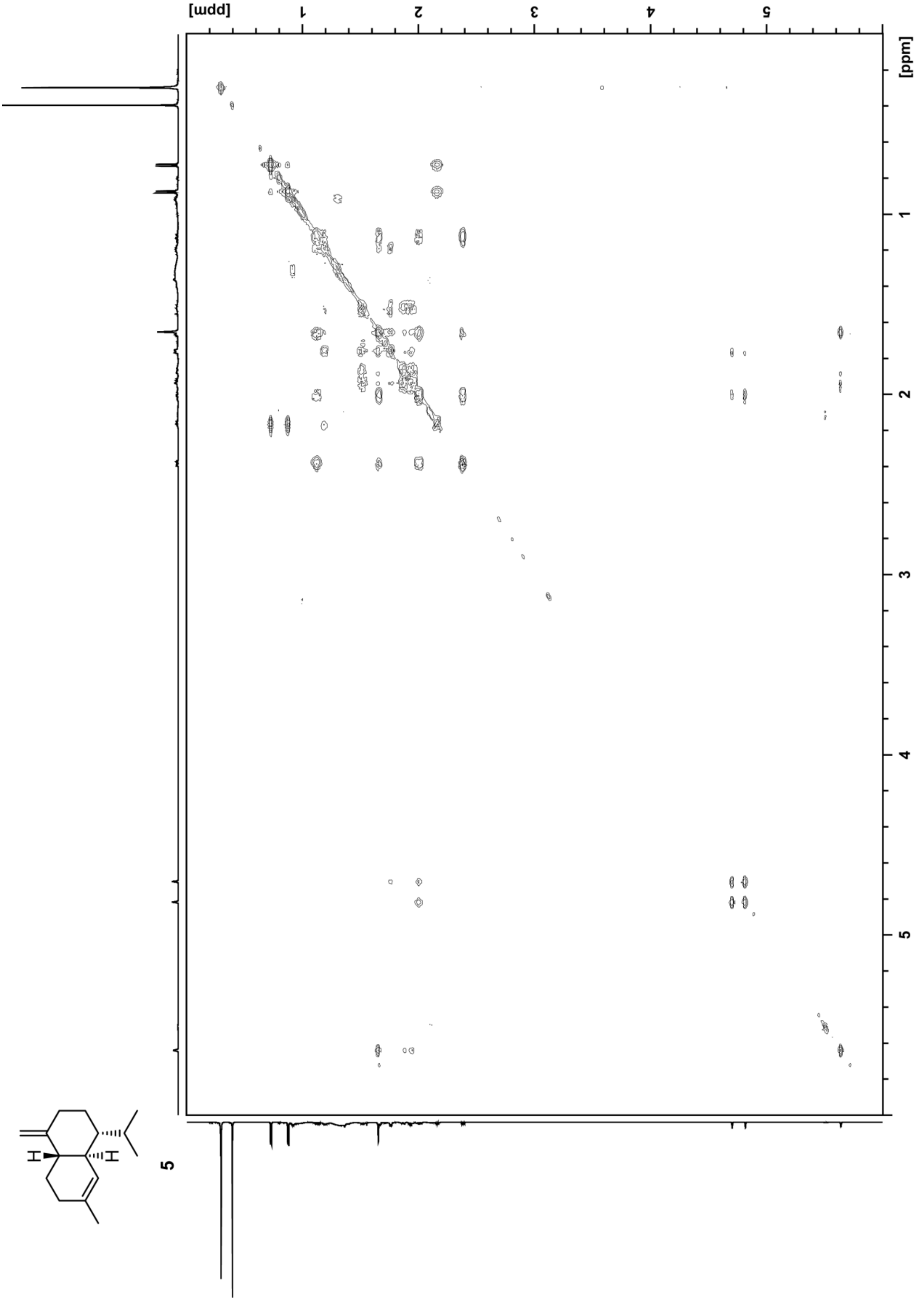

Figure S33: ^{1}H,^{1}H-COSY spectrum of **5**.

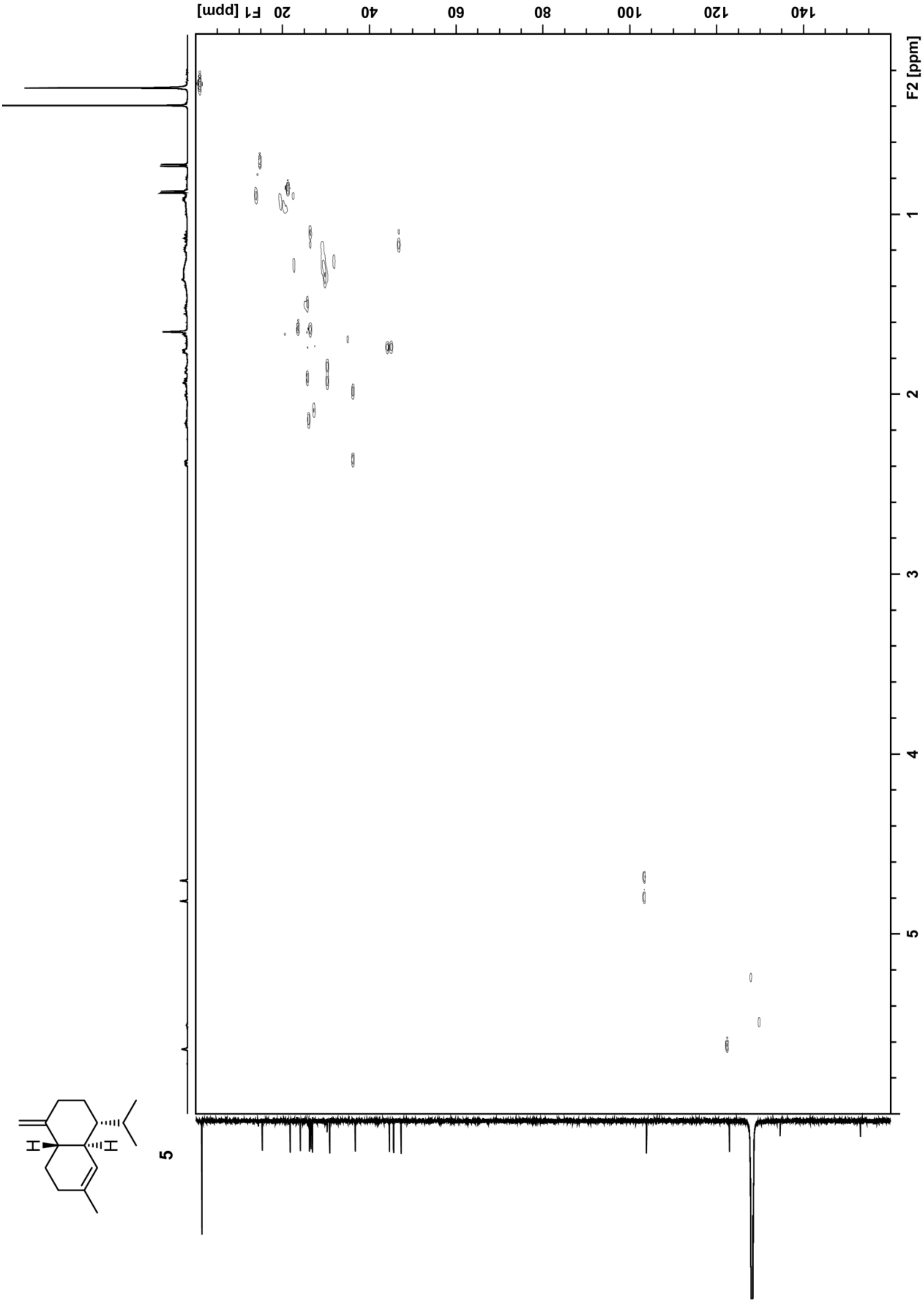

Figure S34: HSQC spectrum of **5**.

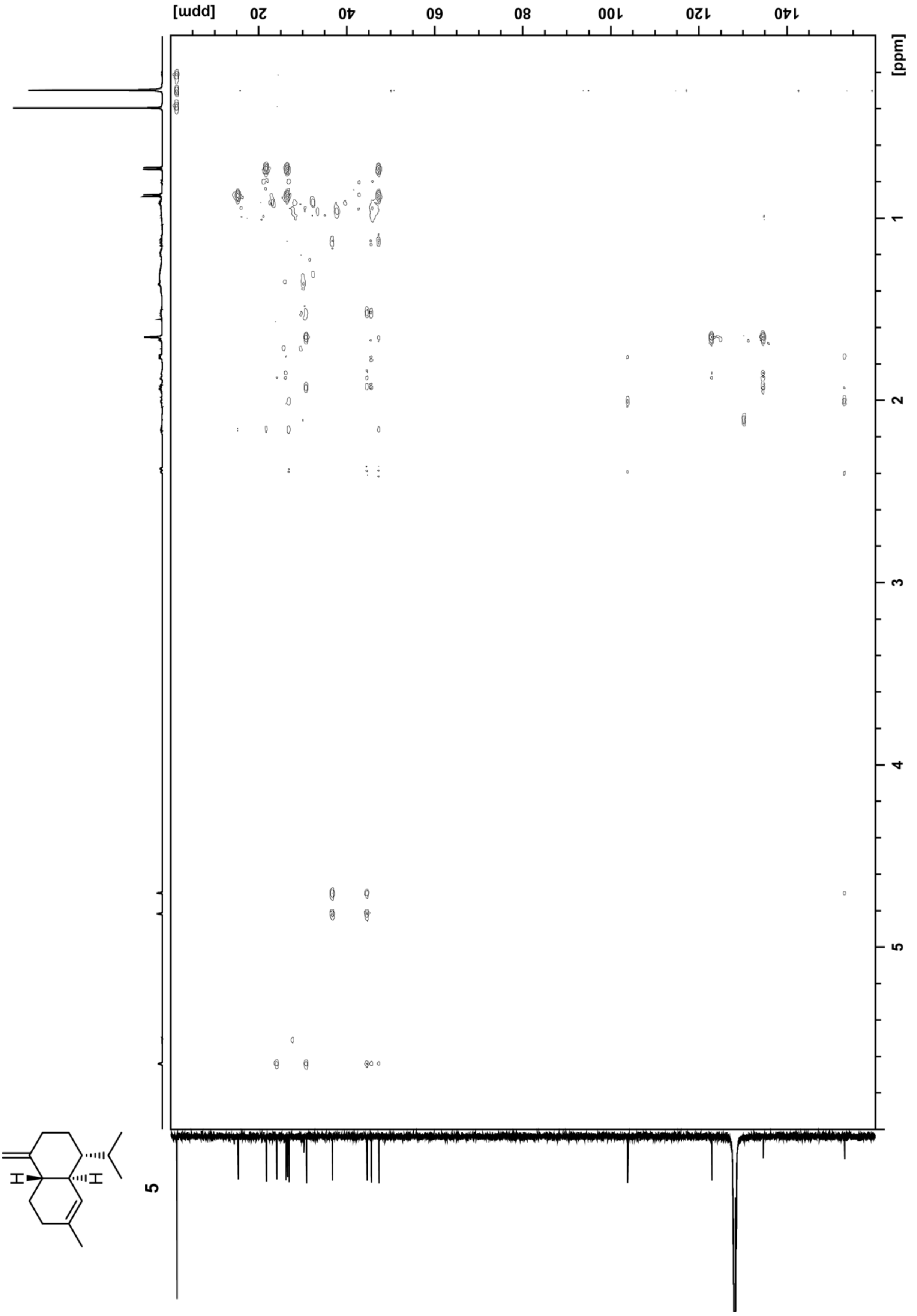

Figure S35: HMBC spectrum of **5**.

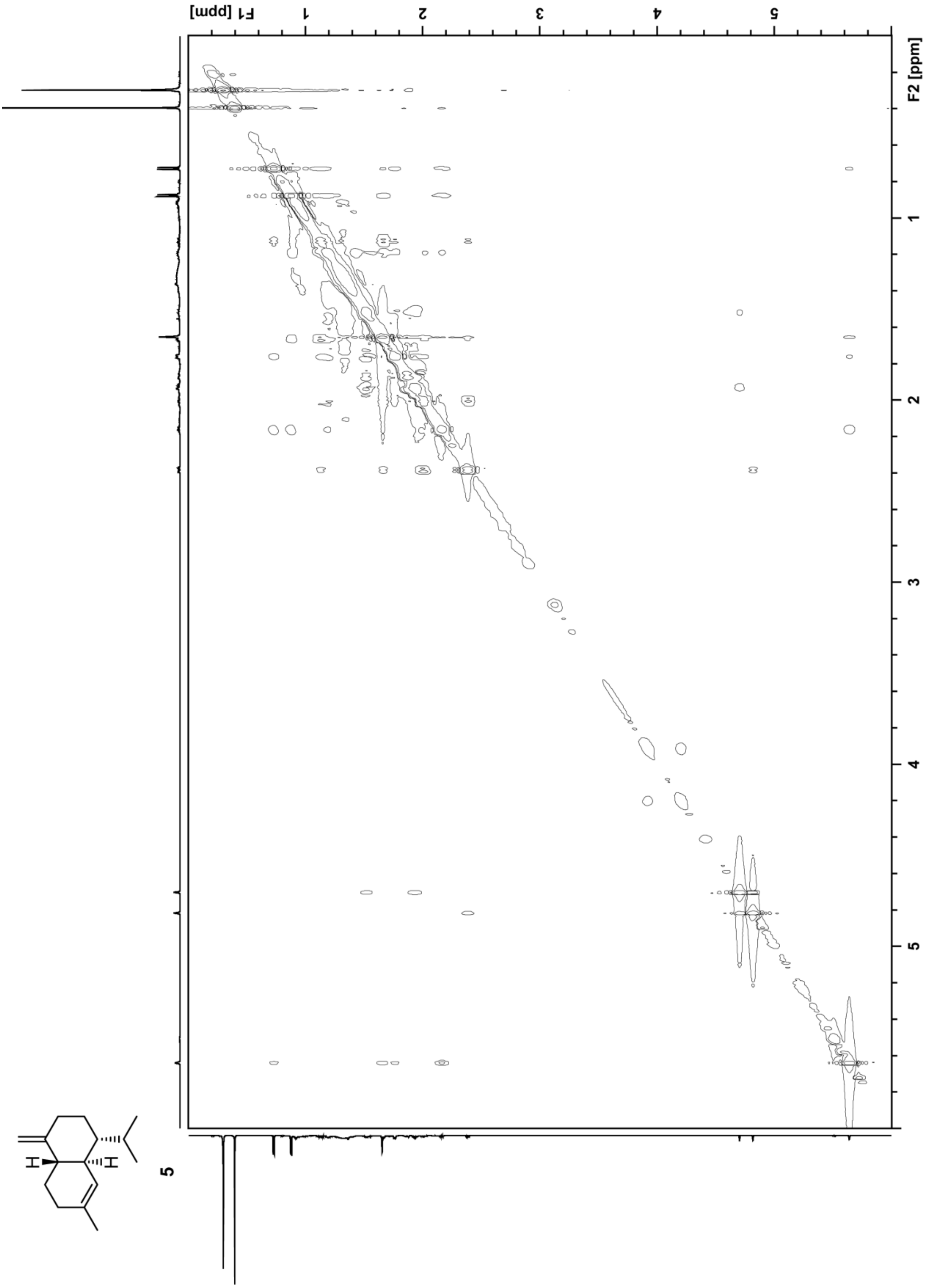

Figure S36: NOESY spectrum of **5**.
